# Mei5–Sae3 stabilizes both active and inactive forms of Dmc1 filaments independently of its impact on ATP hydrolysis

**DOI:** 10.1093/nar/gkaf1085

**Published:** 2025-11-06

**Authors:** Yuen-Ling Chan, Diedre Reitz, Brian Budke, Phoebe A Rice, Douglas K Bishop

**Affiliations:** Department of Radiation and Cellular Oncology, Department of Molecular Genetics and Cell Biology, University of Chicago, Chicago, IL 60637, United States; Committee on Genetics, Genomics, and Systems Biology, University of Chicago, Chicago, IL 60637, United States; Department of Microbiology and Molecular Genetics, University of California, Davis, Davis, CA 95616, United States; Department of Radiation and Cellular Oncology, Department of Molecular Genetics and Cell Biology, University of Chicago, Chicago, IL 60637, United States; Department of Biochemistry and Molecular Biology, University of Chicago, Chicago, IL 60637, United States; Department of Radiation and Cellular Oncology, Department of Molecular Genetics and Cell Biology, University of Chicago, Chicago, IL 60637, United States; Committee on Genetics, Genomics, and Systems Biology, University of Chicago, Chicago, IL 60637, United States

## Abstract

In budding yeast, Dmc1’s recombinogenic activity is controlled by the meiosis-specific heterodimer Mei5–Sae3. Mei5–Sae3 is required for assembly of Dmc1 at sites of meiotic DNA double-stranded breaks. Here, we report Mei5–Sae3 can stabilize Dmc1 filaments in both the active and inactive allosteric conformations depending on the nucleotide cofactor supporting filament formation. Mei5–Sae3 specifically stabilizes the active filament form without inhibiting ATP hydrolysis, in contrast to high concentrations of calcium, AMP–PNP, and the E157D mutation in Dmc1, each of which promotes Dmc1 filament stability by processes that include blocks to ATP hydrolysis. Mei5–Sae3 increases Dmc1 ATP hydrolysis by a mechanism that could be a cause of active filament stabilization or a secondary and inconsequential effect of active filament stabilization. Mei5–Sae3 can also stabilize filaments in the inactive conformation with ADP as a cofactor. These results show that Mei5–Sae3’s filament stabilization activity does not fully depend on alteration of the hydrolytic cycle. We also show Dmc1–E157D, a gain-of-function protein that bypasses the requirement for Mei5–Sae3 *in vivo*, is defective in ATPase activity and stabilizes the active form of Dmc1 filaments as predicted by previous observations. Hence, Dmc1’s homology search and strand exchange activities do not depend on its ability to hydrolyze ATP.

## Graphical Abstract

**Graphical Abstract:**
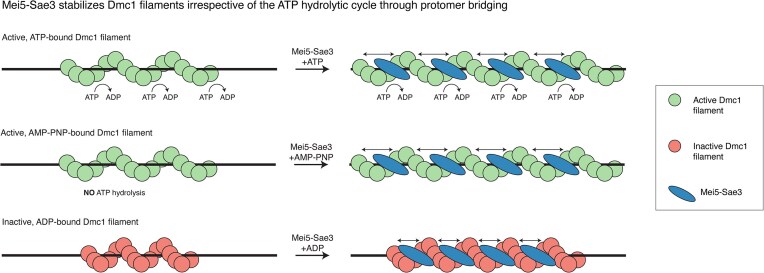

## Introduction

During the first meiotic division, homologous recombination (HR) physically links the homologous chromosomes to one another to facilitate their reductional segregation [1]. The enzyme responsible for carrying out meiotic recombination in most eukaryotes is Dmc1, a RecA homolog [2]. Following deliberate double-stranded break (DSB) formation by the meiosis-specific transesterase Spo11, the 5′ strands of double-stranded DNA (dsDNA) ends are resected to generate 3′ single-stranded DNA (ssDNA) tracts [3]. Initially, these ssDNA overhangs are coated by the ssDNA-binding protein replication protein A (RPA). RPA protects the ssDNA from degradation and prevents secondary structure formation [4]. Dmc1 then displaces RPA and loads onto the ssDNA tracts, forming a helical nucleoprotein filament. The process of filament formation requires the Dmc1 accessory factors Mei5–Sae3 in budding yeast [5, 6]. Using the ssDNA on which it is bound as a guide, Dmc1 conducts a genome-wide search for a homologous region of dsDNA. Upon identifying a suitable target or donor sequence, Dmc1 promotes strand invasion and the formation of Watson–Crick basepairs between the ssDNA to which it is bound and the complementary strand of the target duplex DNA identified by the search, forming a strand invasion product named the D-loop.

Recombinase RecA and its eukaryotic homologs, Rad51 and Dmc1, form active helical filaments that share several properties. The filaments are right-handed, and, when in the active form, have a pitch of ∼10 nm [7–11]. Protomers within this filament bind ∼3 nucleotides, with one turn consisting of six protomers. RecA and its homologs bind ATP and are DNA-dependent ATPases. The ssDNA within the filaments is held in an extended conformation that disrupts stacking interactions between groups of three bases [12]. ATP binding, but not hydrolysis, is required for DNA binding and assembly of the active form of the nucleoprotein filament that supports strand exchange [13–15]. RecA ATP hydrolysis is necessary to clear recombinase protomers from the heteroduplex product of strand exchange and to promote disassembly of off-pathway (dead-end) dsDNA complexes [15–17]. Hydrolysis of ATP to ADP by RecA protomers is associated with an allosteric conversion of the filament from the high DNA-binding affinity, active filament form with a pitch of 10 nm to a lower affinity, inactive form with a pitch of ~8 nm [18, 19]. Notably, a divalent cation is required at the ATPase site for ATP hydrolysis. Usually, this divalent cation is magnesium (Mg^2+^), which is present at much higher levels *in vivo* than calcium (Ca^2+^) [20, 21]. Substituting Ca^2+^ for Mg^2+^ stimulates Rad51/Dmc1 strand exchange activity by inhibiting its ATPase activity [22–26].

The overall filament structures formed by RecA, Rad51, and Dmc1, are conserved [8–10, 19, 27]. However, the mechanism through which ATP hydrolysis is used to promote recombinase-DNA binding dynamics for the eukaryotic RecA homologs Rad51 and Dmc1 is somewhat more complicated than for RecA [28]. Rad51 and Dmc1 have comparatively low rates of ATP hydrolysis, and protomers in the collapsed ADP-bound filament form tend to remain bound to DNA [29, 30]. Thus, they require the translocases Rad54 and Rdh54 (a.k.a. Tid1) to remove them from dsDNA [17, 31, 32]. Importantly, RecA and Rad51 mutants specifically defective in ATP hydrolysis retain the ability to promote strand exchange *in vivo* and *in vitro* [16, 23, 33–39]. Correspondingly, the D317K mutation in human DMC1 has reduced DNA-dependent ATPase activity but is proficient for strand exchange *in vitro* [40]. Though it is reasonable to infer that ATP hydrolysis will also be dispensable for Dmc1-mediated recombination in cells, this has never been formally demonstrated with a validated Dmc1 ATPase mutant.

The eukaryotic recombinase accessory protein family here referred to as the Swi5–Sfr1 family is critical for HR. Members of this family include Mei5–Sae3 in *Saccharomyces cerevisiae*, or budding yeast, Swi5–Sfr1 in *Schizosaccharomyces pombe*, or fission yeast, Swi5–Sfr1 in mouse, and MEI5–SWI5 in humans [41]. Note that we have chosen to refer to the two heterodimers as Mei5–Sae3 and Swi5–Sfr1 as is the convention in the budding and fission yeast fields, respectively, although Mei5 is a homolog of Sfr1, and Sae3 is a homolog of Swi5. Swi5–Sfr1 family members are required for DNA break-dependent recombinase focus formation *in vivo*, indicating a role in initiating or stabilizing recombinase filaments [5, 6, 42, 43].

Though much is known about the biochemical properties of Swi5–Sfr1 family members, the mechanism through which they promote the assembly and/or stability of filaments for their cognate recombinase is unclear. Biochemical studies have provided evidence that Swi5–Sfr1 and Mei5–Sae3 are involved in filament formation with and without RPA and shown that Mei5–Sae3 leads to more continuous Dmc1 filaments [44, 45]. In addition, fission yeast Swi5–Sfr1 has been shown to increase Rad51 and Dmc1 filament stability by decreasing dissociation [44, 46]. Finally, the fission yeast and mouse Swi5–Sfr1 stimulate Rad51 ATPase activity, though how this relates to Swi5–Sfr1’s role in promoting Rad51 filament formation/stability is unclear [47, 48].

Importantly, several *in vitro* studies imply that Swi5–Sfr1’s ability to promote Rad51/Dmc1 filament formation and/or stability is dependent on ATP hydrolysis. Flow linear dichroism spectroscopy showed Swi5–Sfr1 causes the DNA bases in Rad51 filaments to become perpendicularly aligned to the filament axis [49]. This coplanar alignment is believed to represent the active form. When Ca^2+^ was used in place of Mg^2+^ to assemble *S. pombe* Rad51 filaments a similar effect was observed, but this effect was not additive with that of Swi5–Sfr1 [49]. These structural analyses suggest that limiting or preventing ATP hydrolysis by addition of Ca^2+^ has a similar effect on the Rad51 filament to that of Swi5-Sfr1, yet the mechanism of action of Swi5-Sfr1 must be different, since Swi5-Sfr1 and Ca^2+^ have opposite effects on hydrolysis [22–26, 47, 48, 50]. Working with purified mouse proteins, Chi and colleagues concluded that ssDNA-dependent stimulation of RAD51 ATPase activity by Swi5-Sfr1 stabilizes the active, ATP-bound filament form indirectly by promoting ADP release [48].

We previously identified a putative ATPase-defective mutant of Dmc1, Dmc1–E157D, as a *DMC1* allele that bypasses Mei5–Sae3 [51]. While Dmc1–E157D-mediated recombination has notable defects, including elevated ectopic and inter-sister recombination, it nevertheless forms high levels of recombination products and produces viable progeny. These results strongly suggested that the critical impact of Mei5–Sae3 on Dmc1 involves stabilizing the active filament form.

Herein, we show that Mei5–Sae3 stabilizes Dmc1 filaments in the active allosteric form and accelerates ATP hydrolysis specifically at low, physiological levels of calcium ions. However, the ability of Mei5–Sae3 to stabilize Dmc1 nucleoprotein filaments can also be observed in the presence of ADP, which promotes formation of Dmc1 filaments in the inactive allosteric form, as well as in the presence of the nonhydrolyzable ATP analog AMP–PNP, which stabilizes the active filament form. Thus, Mei5–Sae3 stabilizes both active and inactive allosteric filament forms depending on the nucleotide cofactor and can exert this effect without an associated alteration of the ATP hydrolytic cycle. Biochemical analysis of Dmc1–E157D shows it forms active filaments in the absence of Mei5–Sae3 and confirms that it is ATPase defective, indicating that Dmc1’s ATPase activity is not required for its homology search and strand exchange functions *in vivo*.

## Materials and methods

### Recombinant proteins

This study uses an N-terminal 6x-His-tagged version of *Saccharomyces cerevisiae* proteins [36]. *Sc*Dmc1 and its mutant derivative *Sc*Dmc1–E157D were over-expressed and purified from *Escherichia coli* by the method detailed previously [52, 53]. *Sc*Dmc1 was over-expressed from the plasmid pNRB150 and *Sc*Dmc1–E157D from pNRB756. The N-terminal 6x-His-tag has been shown not to affect *Sc*Dmc1 function [39]. The cell lysis buffer P (25 mM sodium phosphate buffer pH 7.5, 1 mM dithiothreitol (DTT), 1 M NaCl, 10% glycerol, and 50 mM imidazole) was modified to include 1 mM ATP and 1 mM Mg^2+^ to reduce ATPase contamination and prevent *Sc*Dmc1 aggregation. *Sc*Mei5–Sae3 was expressed from plasmid pNRB716 with the vector pCOLADuet [54] that harbors the open reading frames (ORFs) of both Mei5 and Sae3; Mei5 has an N-terminal 6x-His-tag. *Sc*Mei5–Sae3 was purified by the same procedure used for *Sc*Dmc1, except multiple rounds of purification using a MonoS ion exchange column were added to eliminate ATPase contamination. RPA was expressed from plasmid p11d-sctRPA in *E. coli* and purified as previously described [55]. All purified proteins were stored at −80°C.

**Figure 1.**
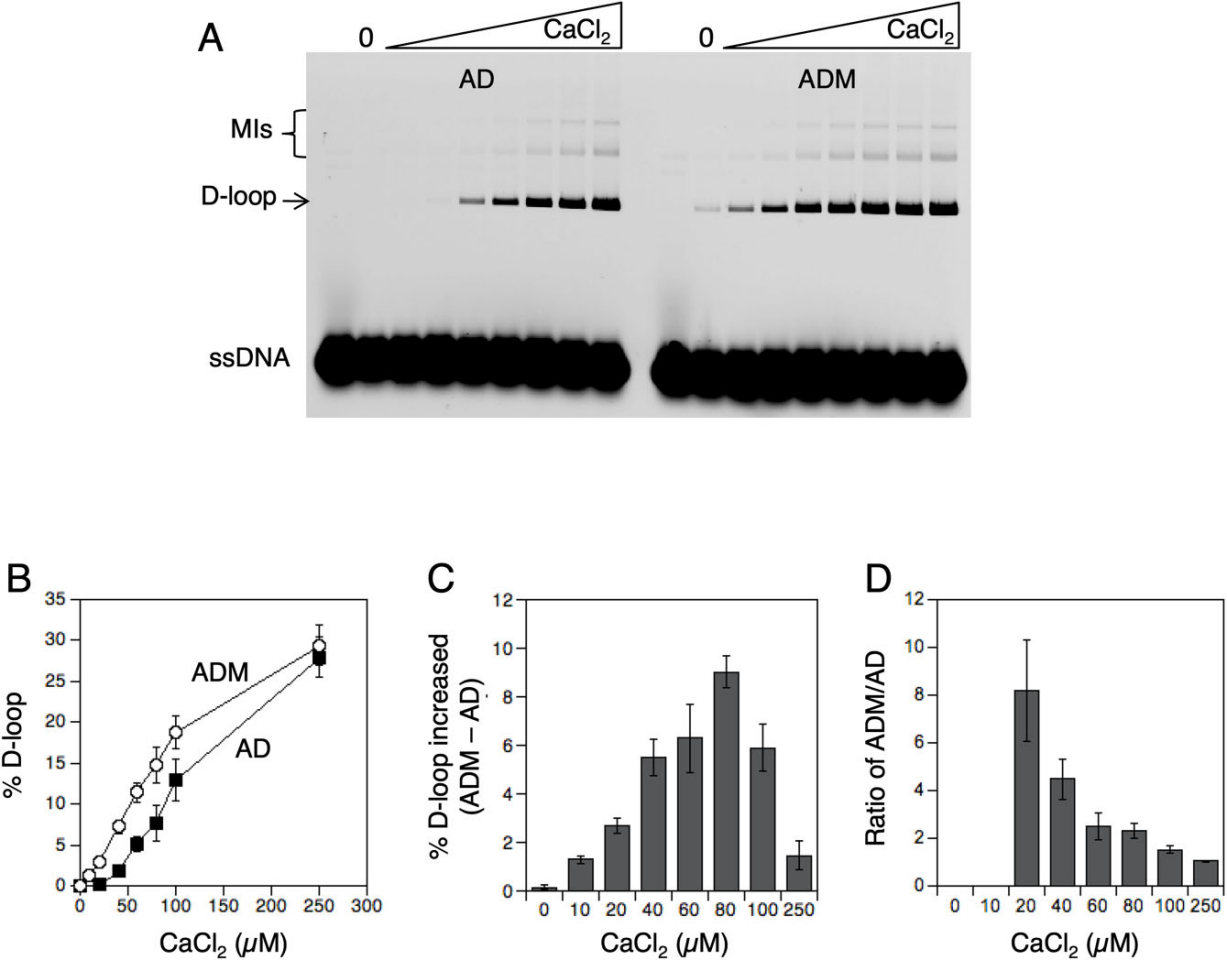
Mei5–Sae3 stimulates the D-loop activity of Dmc1–WT at low Ca^2+^ concentrations. The effect of Mei5–Sae3 on Dmc1–WT was determined using the D-loop assay in the presence or absence of Mei5–Sae3 as indicated, RPA, and varying concentrations of Ca^2+^. (**A**) A representative image of a D-loop gel is shown. Protein acronyms and concentrations are as follows: A = RPA (0.2 µM), D = Dmc1–WT (3 µM), and M = Mei5–Sae3 (0.5 µM), and MI = multi-invasion recombination byproduct. Ca^2+^ concentrations are: 0, 10, 20, 40, 60, 80, 100, and 250 µM. The first lane of each D-loop experimental set is a no protein control. (**B**) D-loop activity is plotted as averages ± SEM (*n* = 3). The stimulation by Mei5–Sae3 is expressed as a difference (**C**) or a ratio (**D**) of the D-loop activity of reactions ADM versus AD ± SEM (*n* = 3). The preparation of Dmc1 used in the experiments described in this figure (which was purified on 22 February 2017) had a somewhat lower specific activity than the preparation of Dmc1 used in generating the other data in the paper (28 May 2019).

Two different preparations of ScDmc1–WT were used. The preparation of Dmc1 used in Fig. 1 (which was purified on 22 February 2017) had a somewhat lower specific activity than the preparation of Dmc1 used in generating other data in the paper (28 May 2019).

### Plasmids, DNA, and radioactive compounds

The supercoiled plasmid pRS306 was purified by cesium chloride equilibrium density gradient centrifugation [36]. The 90 nt-ssDNA (complementary to bp 764–853 in pRS306) was either unlabeled or 5′-labeled with a fluorescent dye Quasar670 and synthesized by LGC Biosearch Technologies. The fluorescent 563 nt-ssDNA (complementary to bp 306–868 in pRS306) was generated by asymmetric polymerase chain reaction (PCR) using a 5′-fluorescent Quasar670 labeled primer in 50-fold excess of the unlabeled 3′-primer [52]. The PCR products were separated in a composite native gel of 2.5% polyacrylamide and 0.5% agarose in the Tris-borate buffer (TBE). The fluorescent ssDNA fragments were excised and eluted from the gel by electro-elution using Tris acetate buffer (TAE).

The unlabeled 2627 nt-ssDNA used in reactions for electron microscopy (EM) was produced and isolated from pRS306 circular ssDNA. Briefly, two EcoRI sites were first engineered into the phagemid pRS306 and transformed into JM109 *E. coli* cells. Circular ssDNA was produced and isolated using M13K07 helper phage (NEB) following the manufacturer’s instructions. The circular ssDNA was annealed to two oligo-ssDNAs with EcoRI sites and digested to generate the 2627-nt fragment. The restriction products were separated using a composite native gel of 2.5% polyacrylamide and 0.5% agarose in TBE. The 2627 nt-ssDNA fragment was excised and eluted from the gel by electro-elution using TAE.

Radioactive α-^32^P-ATP (3 000 Ci/mmol) was purchased from Perkin Elmer. The tritium labeled [8–3H]-AMP–PNP (25 Ci/mmol) and [2,8–3H]-ADP (28 Ci/mmol) were custom synthesized by American Radiolabeled Chemicals, Inc.

### D-loop assay

D-loop reactions with fluorescent dye Quasar670-labeled ssDNA were conducted as described [52, 53]. The reaction was pre-staged at either 30°C or 37°C as indicated in figures. First, 0.2 µM RPA and 30 nM (2.7 µM nt) 90 nt-ssDNA was incubated for 5 min in reaction buffer D (25 mM Tris–HCl, pH 7.5, 1 mM DTT, 5 mM Mg^2+^, 3 mM ATP, and 100 µg/ml bovine serum albumin (BSA)) with 70 µM Ca^2+^ or at the concentrations indicated in figures and legends. The concentration of NaCl in the reactions, contributed by storage buffer, is around 50 mM. Next, 3 µM ScDmc1 (or ScDmc1–E157D) was added with or without 0.5 µM Mei5–Sae3 and incubated for 8 min to form nucleoprotein filaments. Strand exchange reactions were initiated by addition of 5 nM plasmid pRS306 (22 µM bp) with or without 0.1 µM Rdh54. After 30-min incubation, sodium dodecyl sulfate (SDS) (1%) and proteinase K (1 mg/ml) were added, followed by a 5-min incubation at 37°C. The deproteinized samples were loaded onto a 0.9% agarose gel and subjected to electrophoresis in TAE buffer. The wet gel was analyzed using direct phosphor-imaging on a GE Amersham Typhon 5 Imager. The intensity of both the D-loop and the unincorporated ssDNA bands were imaged in the linear range. The D-loop yield was expressed as a percentage of input plasmid DNA.

### DNA binding using fluorescence polarization spectroscopy

DNA binding assays by fluorescence polarization (FP) [56] were performed in buffer (25 mM HEPES pH 7.5, 1 mM Mg^2+^, 1 mM ATP, 1 mM DTT, 50 mM NaCl, 100 µg/ml BSA, 0.02% Nonidet-P40, and 70 µM Ca^2+^ or as indicated) containing 3 nM (250 nM-nt) of 84 nt-ssDNA. We used 1 mM Mg^2+^ rather than 5 mM to allow comparison with previous polarization data from our laboratory that is not presented here. Dmc1 concentration was titrated up to 3 µM and Mei5–Sae3 was 0.4 µM or as indicated in figures. Dmc1 was added to the reactions last. The ssDNA was labeled with 5′ Alexa Fluor 488(5′-GGTAGCGGTTGGGTGAGTGGTGGGGAGGGTCGGGAGGTGGCGTAGAAACATGATAGGAATGTGAATGAATGAAGTACAAGTAAA) and was synthesized by IDT. The 50 µl samples were assembled on a polystyrene plate (Corning 384 flat bottom black plate), incubated at 30°C for 20 min, and analyzed in a Tecan Infinite F200 Pro plate reader (Life Sciences). The binding curves were plotted and analyzed using KaleidaGraph software. Apparent dissociation constants (*K*_D_) were derived from binding curves using the same software.

### ATPase activity assay

ATP hydrolysis was analyzed by thin layer chromatography (TLC), which separates α-^32^P-ADP from α-^32^P-ATP. Dmc1 (at either 2 or 3 µM) was incubated together with other proteins indicated at 30°C in buffer (25 mM Tris–HCl, pH 7.5, 1 mM DTT, and 5 mM Mg^2+^) containing 67 nM (6 µM nt) of 90 nt-ssDNA, 100 µM ATP containing 0.04 µCi α-^32^P-ATP and 70 µM Ca^2+^. Dmc1 was added last to a reaction to initiate ATP hydrolysis. The concentration of NaCl in the reactions from diluted proteins was ~50 mM. At the indicated time intervals, a 2 µl aliquot was removed and mixed with 0.5 µl of 10 N formic acid to terminate ATP hydrolysis; then a 2 µl of this mixture was applied onto a thin-layer plate (PEI cellulose F from Merck). Chromatography was done in a buffer containing 0.5 M LiCl and 1 M formic acid. When done, the TLC plate was dried and exposed to an imaging plate and phosphorimaging was done on a GE Amersham Typhon 5 Imager.

### Nitrocellulose filter assay for nucleotide binding

A reaction of 10 µl with indicated proteins (3 µM Dmc1, or 3 µM Dmc1–E157D, with or without 1 µM Mei5–Sae3) and 67 nM (6 µM nt) of 90 nt-ssDNA) was assembled in a buffer containing 25 mM Tris–HCl, pH 7.5, 1 mM DTT, 5 mM Mg^2+^, 70 µM Ca^2+^, and 100 µM of α-^32^P-ATP. The order of addition of ssDNA, α-^32^P-ATP, and Dmc1 (or Dmc1–E157D) to a reaction premixture containing all other components is indicated in the figure and its legend. The samples were incubated at 30°C for 5 min before placing on ice. A pre-cut nitrocellulose filter (Osmonics Inc.) was pre-soaked in reaction buffer and was placed on top of a Millipore fritted glass filter support (4 cm diameter), a vacuum was applied to draw liquid through the filter. For each reaction, 3 µl drop aliquots of a given sample were spotted at the same position on the filter three times in succession (3 × 3 µl per sample; a filter can accommodate 15 drops). After sample application, the filter was washed twice with 5 ml reaction buffer, dried, and analyzed by phosphorimaging. The binding curves were plotted and analyzed using KaleidaGraph.

### Measuring release of 3H-ADP or 3H-AMP–PNP from Dmc1 filament by Mei5–Sae3

Reaction mixtures of total volume 10 µl in buffer (25 mM Tris–HCl, pH 7.5, 1 mM DTT, 5 mM Mg^2+^, and 70 µM Ca^2+^) containing 100 nM of 90 nt-ssDNA (9 µM nt) and 3 µM 3H-ADP (or 3H-AMP–PNP) were mixed at room temperature for 5 min. Dmc1 (3 µM) was then added to the premixture and incubated at 30°C for 30 min before the addition of varying amount of Mei5–Sae3 (0.5, 1, or 2 µM), followed by a 5-min incubation. The samples were passed through nitrocellulose filter as detailed in filter assay described above. The filter that retained the tritium labeled protein–DNA complexes was first incubated in 300 µl of water in a vial for 30 min before adding 4 ml scintillation fluid. The radioactivity in filters was counted in a Packard Tri-Carb Liquid Scintillation Counter (by GMI Trusted Laboratory Solutions).

### Visualization of nucleoprotein filaments by electron microscopy

A 10 µl reaction containing 0.2 µM RPA and 2.3 nM 2627 nt-ssDNA (6 µM nt) was first incubated for 5 min at 37°C in D-loop buffer D containing 70 µM Ca^2+^ (or as indicated in figure legends) without BSA. Next, 3 µM *Sc*Dmc1 (or *Sc*Dmc1–E157D) and/or Mei5–Sae3 (2 µM) was added to the RPA + ssDNA mixture and incubated at 30°C for 10 min followed with 37°C for 5 min to form nucleoprotein filaments before storing on ice. A 5 µl aliquot was spread onto a carbon coated grid and negatively stained with 1% uranyl acetate. The grids were examined, and images were recorded with a GATAN digital camera at ×25 000 using a FEI Tecnai G2 F30 300 kV Super Twin Electron Microscope in the University of Chicago Advanced Electron Microscopy Core Facility. EM images of filaments were collected by facility staff without bias or knowledge of which sample was which. The contour length of the filaments was determined using the line tool of ImageJ software and calibrated via the microscope generated size bar. The filament length in nm was converted to the corresponding nucleotide length using 1 nm = 1.8 nt based on the analysis of *Sc*Dmc1 filaments which on average has 6.5 protomers per turn with a helical pitch of ∼106 Å [24]. Regions containing high density of overlapping filaments were excluded from analysis.

### Analysis of Dmc1 filament-bound nucleotides

Reactions (20 µl) containing a combination of 6 µM nt ssDNA (90 nt), 100 µM α-^32^P-ATP, with or without 1 µM Mei5–Sae3 were first assembled in buffer, and 3 µM Dmc1 was added last to initiate ATP hydrolysis according to the procedures described above for ATP hydrolysis. The reactions were then incubated at 30°C for 40 min. Aliquots (5 × 3 µl) of reaction samples were dotted on a nitrocellulose filter and unbound nucleotide removed by washing the filter with 2 × 5 ml binding buffer. The filter containing bound filaments were cut into small pieces and soaked in 100 µl elution buffer (25 mM Tris–HCl, pH 7.5, 5 mM EDTA, 1% SDS, and 1 mg/ml protease K); incubated at 37°C for 20 min with agitation. Aliquots of the eluent (5 × 3 µl per reaction) were spotted on TLC plates and subjected to chromatography for separation of α-^32^P-ADP from α-^32^P-ATP. The TLC plate was analyzed by phosphoimaging and data analyzed using KaleidaGraph.

## Results

### Stimulation of Dmc1 by Mei5–Sae3 increases as Ca^2+^ concentration is lowered

We previously studied Mei5–Sae3 activity under two different sets of biochemical conditions. In the first study, we identified conditions under which a preparation of the ssDNA binding protein RPA inhibited the activity of Dmc1 and found that Mei5–Sae3 could suppress that inhibition [57]. In that study, Ca^2+^ was used at a high, nonphysiological concentration and no Mg^2+^ was present. In a more recent study, we developed a biochemical reconstitution system for Dmc1 D-loop activity that includes Dmc1 and its five known accessory proteins [53]. In the reconstitution study, protein concentrations were optimized for D-loop yield. At optimal Dmc1 concentration, RPA stimulated, rather than inhibited Dmc1’s activity, as did the other four accessory proteins including Mei5–Sae3. Although Mei5–Sae3 enhanced D-loop yield in the reconstitution system, the enhancement was quite modest (1.2-fold). This modest enhancement is in dramatic contrast to the strict requirement for Mei5–Sae3 to support the activity of wild-type Dmc1 (Dmc1–WT) *in vivo* [5, 6, 58]. Among the many differences between *in vivo* and reconstitution conditions is the fact that the reconstitution studies were carried out using 250 µM Ca^2+^, a concentration >3-fold higher than the previously reported total concentration of Ca^2+^ in yeast cells of 70 µM [20, 59]. In addition, most previous studies of Mei5–Sae3 homologs showed that their ability to stimulate their cognate recombinases was not seen when Ca^2+^ was used in place of Mg^2+^ [48, 49, 60, 61]. It was therefore of interest to determine if Dmc1–WT D-loop reactions show a greater dependency on Mei5–Sae3 at lower, more physiological Ca^2+^ concentrations. For these titration experiments, all reactions contained 5 mM Mg^2+^ in addition to the indicated level of Ca^2+^. We found that reducing Ca^2+^ levels below 250 µM lowered total D-loop yields but increased the dependency of the yield on Mei5–Sae3 (Fig. 1). At the physiological level of 70 µM Ca^2+^, we observed ~2.5-fold more D-loop product in reactions containing Mei5–Sae3 than in reactions without it. Thus, the ability of Mei5–Sae3 to stimulate Dmc1’s activity is greater at physiological Ca^2+^ concentrations; high Ca^2+^ levels bypass the requirement for Mei5–Sae3.

### Dmc1–E157D, an ATPase mutant that bypasses Mei5–Sae3, is unstable *in vitro* and requires high levels of Ca^2+^

**Figure 2.**
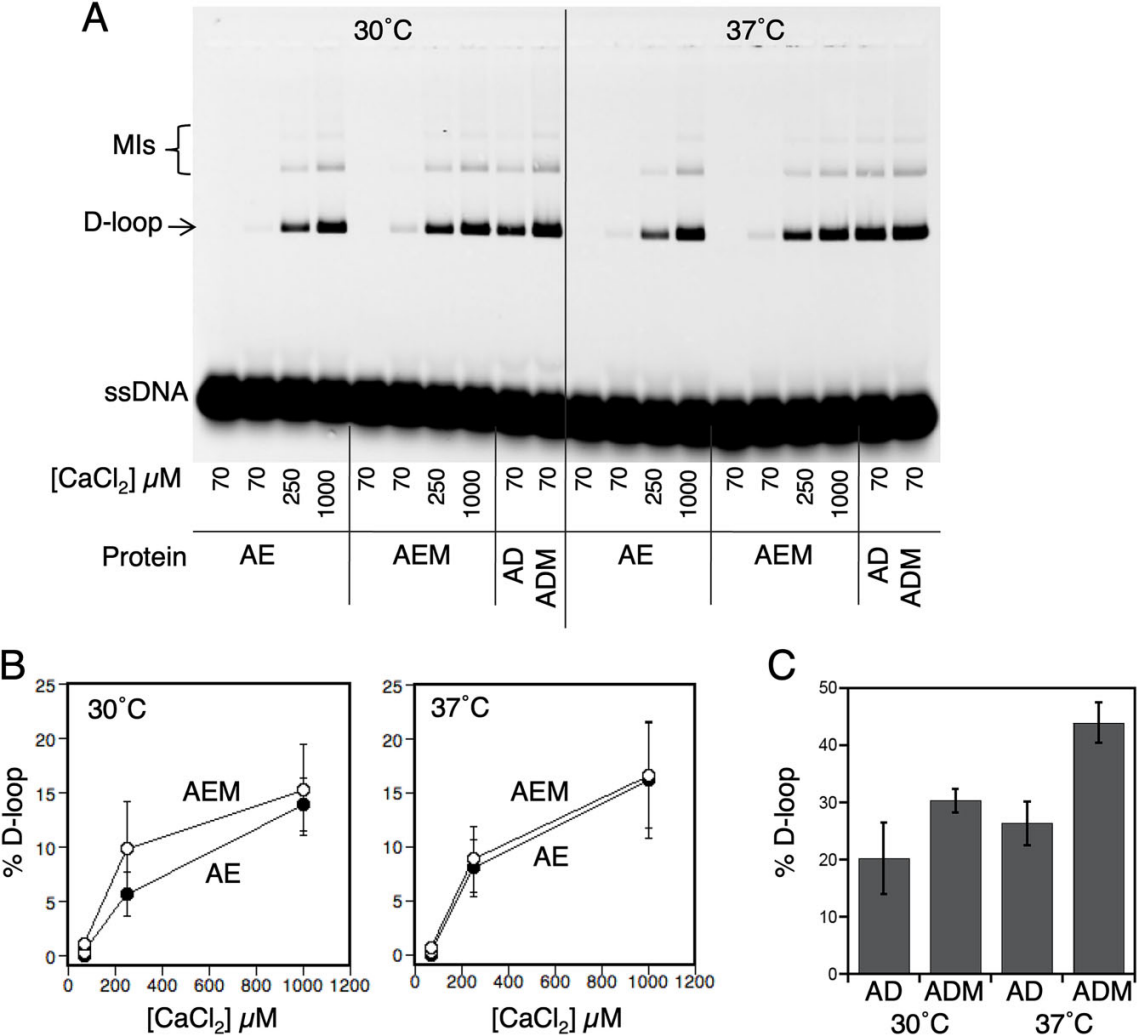
Mei5–Sae3 does not stimulate the D-loop activity of Dmc1–E157D. The effect of Mei5–Sae3 on Dmc1–E157D was determined using the D-loop assay at 30°C and 37°C with various concentrations of Ca^2+^. (**A**) A representative image of a D-loop gel is shown. The first lane of each section is a negative control with no Dmc1–E157D. (**B**) Plot of Dmc1–E157D D-loop activity with and without Mei5–Sae3. (**C**) Positive control was using Dmc1–WT with 70 µM Ca^2+^. The protein acronyms and concentrations are: A = RPA (0.2 µM), D = Dmc1–WT (3 µM), E = Dmc1–E157D (3 µM), and M = Mei5–Sae3 (0.5 µM). Ca^2+^ concentrations are as indicated. D-loop activity is plotted as averages ± SEM (*n* = 3). The preparation of Dmc1 used here and all other figures was different than that used in Fig. 1 and has slightly higher specific activity in D-loop assays.

We also examined the D-loop activity of a mutant form of Dmc1, Dmc1–E157D, which bypasses the genetic requirement for Mei5–Sae3 *in vivo* [51]. Given the mutant protein’s ability to support wild-type levels of meiotic recombination in the absence of Mei5–Sae3, we expected it to have at least as much activity as the wild-type protein in the D-loop assay. We further expected that Dmc1–E157D’s activity would not be stimulated by Mei5–Sae3. We tested the wild-type and mutant proteins’ activities using a 90 nt-ssDNA substrate. Although we detected normal levels of activity for the mutant protein, that activity was dependent on high Ca^2+^ concentrations (Fig. 2A and B). Furthermore, while the activity of Dmc1 decreased with increasing Ca^2+^ concentrations between 0.25 and 2.0 mM, the activity of Dmc1–E157D increased in that range (Supplementary Fig. S1A).

Given the different calcium requirements for Dmc1 versus Dmc1–E157D, we wanted to determine if the mutant protein displayed other properties that differed from its wild-type counterpart. We compared the two proteins’ stability by incubating the proteins in buffer without DNA before adding the additional reaction components (Supplementary Fig. S1B). We pre-incubated Dmc1–WT or Dmc1–E157D in reaction buffer for 30 min at 37°C before adding the other reaction components. Control experiments without pre-incubation were used to assess the loss of activity caused by pre-incubation. While the wild-type protein produced nearly equivalent levels of D-loops with or without pre-incubation (24 ± 3% versus 28 ± 1%), Dmc1–E157D formed 2.4-fold fewer D-loops when it was pre-incubated in reaction buffer as compared to without (10 ± 1% versus 24 ± 7%). Thus, purified Dmc1–E157D is less stable than wild-type Dmc1 if pre-incubated at 37°C in the absence of DNA.

In a second test of the stability of Dmc1–E157D, we compared both DNA binding and D-loop activities of the mutant protein at 30°C and 37°C. For measurement of DNA binding, we used a conventional FP assay using a fluorescently tagged 84-nt ss-oligonucleotide as DNA. We found that the DNA binding activity of Dmc1–E157D displayed clear temperature sensitivity; the DNA binding activity of Dmc1–E157D was comparable to that of Dmc1–WT at 30°C but was substantially lower at 37°C (Supplementary Fig. S2). It is interesting to note that when assayed at 50 mM NaCl, 70 μM Ca^2+^ Dmc1–E157D displays similar binding activity relative to the wild-type protein at 30°C (Supplementary Fig. S2), but its D-loop activity is still defective (Fig. 2). This suggests that Dmc1–E157D’s requirement for high concentrations of Ca^2+^ in D-loop formation reflects either an alteration in a quality of DNA binding not detected by FP and/or a defect at a different stage of D-loop formation such as homology recognition.

Since Dmc1–E157D bypasses the requirement for Mei5–Sae3 *in vivo*, we hoped to test if Dmc1–E157D’s D-loop activity could be stimulated by Mei5–Sae3. Unfortunately, the fact that Mei5–Sae3 has no stimulatory activity at high Ca^2+^ concentrations limited us to testing the impact of Mei5–Sae3 under conditions where Dmc1–E157D has very little activity. Although the mutant protein forms a detectable level of D-loops with 70 µM Ca^2+^ at 30°C (Fig. 2A), the reproducibility of yield was not sufficient to provide a reliable test of whether Mei5–Sae3 significantly improves Dmc1–E157D D-loop activity. The altered Ca^2+^ requirement, and other defects of purified Dmc1–E157D, including additional defects described below, limit its utility in mechanistic studies of the normal mechanism of Mei5–Sae3 mediated Dmc1 stimulation.

### Mei5–Sae3 enhances Dmc1’s DNA binding activity

**Figure 3.**
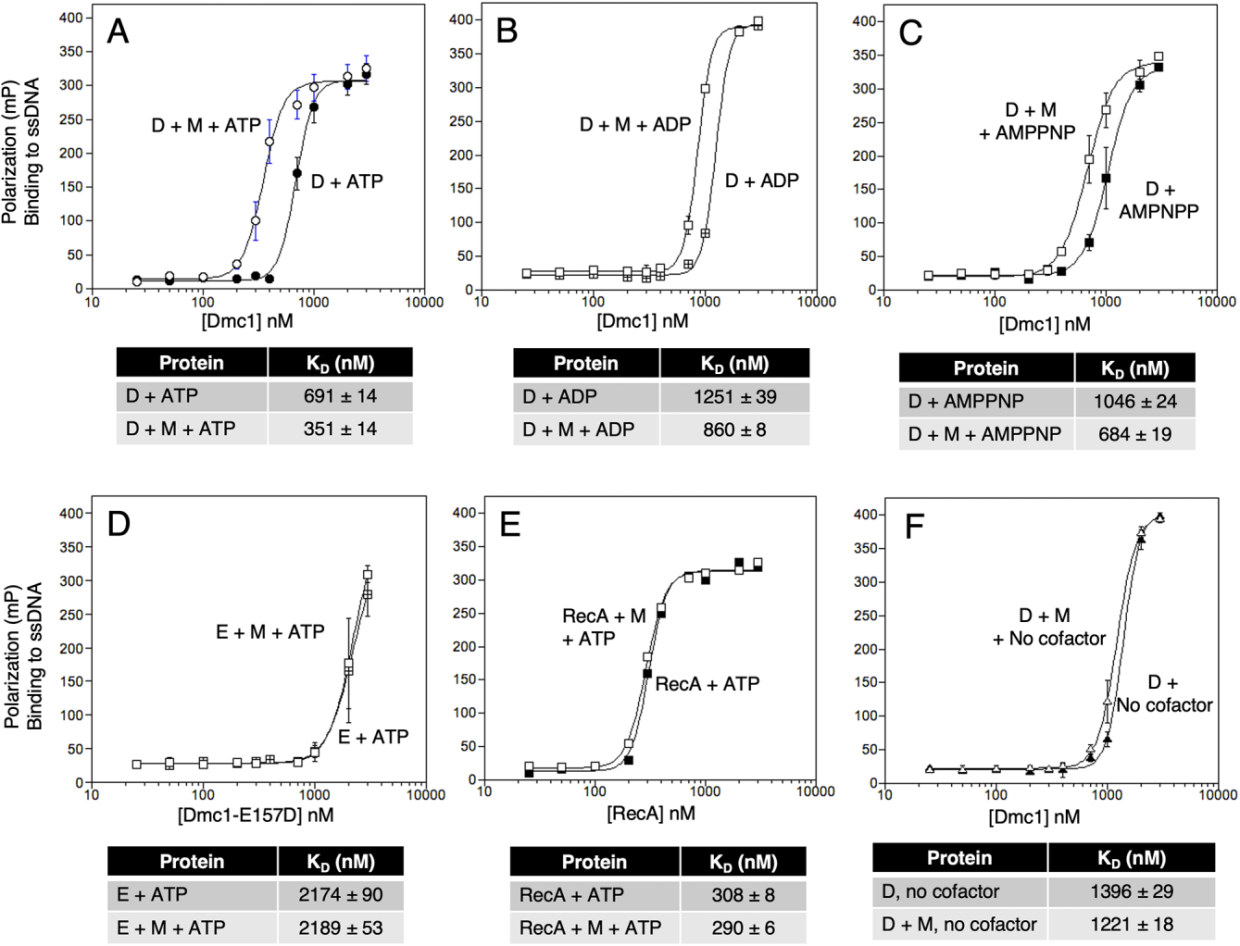
Mei5–Sae3 requires ATP, ADP, or AMP–PNP to enhance Dmc1’s affinity for ssDNA. Binding was determined by FP. The apparent dissociation constant (*K*_D_) of Dmc1 binding to ssDNA was determined from the plots. Dmc1–WT (abbreviated D), Dmc1–E157D (abbreviated E), and Mei5–Sae3 (abbreviated M) all bind ssDNA. At 400 nM Mei5–Sae3, the FP signal was at background levels (Supplementary Fig. S3A); thus this concentration was chosen to be used in subsequent ssDNA-binding reactions with Dmc1. (**A–C**) Mei5–Sae3 enhances the binding of Dmc1 to ssDNA with either ATP or ADP or AMP–PNP. Mei5–Sae3 does not impact ssDNA binding by Dmc1–E157D (**D**) nor the *E. coli* homolog, RecA (**E**). (**F**) Mei5–Sae3 is unable to stimulate Dmc1–WT without nucleotide cofactor.

Because Mei5–Sae3 is required for Dmc1–WT to form foci at sites of DSBs *in vivo*, it was of interest to determine if the impact of Mei5–Sae3 on Dmc1’s D-loop activity at physiological Ca^2+^ concentration was associated with an increase in Dmc1’s DNA binding activity [5, 6, 23, 53]. Analysis of the impact of Mei5–Sae3 on Dmc1’s DNA-binding affinity is complicated by the fact that Mei5–Sae3 itself binds DNA [57, 62]. For this reason, we titrated Mei5–Sae3 and NaCl to identify conditions under which Mei5–Sae3 did not give any signal in the polarization assays (Supplementary Fig. S3A). The conditions we identified that met this criterion were 400 nM Mei5 Sae3 and 100 mM NaCl. Using these conditions, we titrated Dmc1with and without Mei5–Sae3. The Ca^2+^ concentration was fixed at 70 µM and ATP was used as the nucleotide cofactor. In this experiment, we found Mei5–Sae3 reduces the apparent dissociation constant (*K*_D_) of Dmc1–WT for ssDNA ~2.0-fold (Fig. 3A) indicating that Mei5–Sae3 enhances Dmc1 DNA binding activity. We repeated the FP analysis with ADP and the non-hydrolysable ATP analog AMP–PNP in place of ATP and found that Mei5–Sae3 also reduced Dmc1’s *K*_D_ under both conditions, although the reduction was less than that with ATP: ~1.5-fold for both ADP and AMP–PNP (Fig. 3B and C). Thus, Mei5–Sae3 enhances the binding affinity of Dmc1 in the presence of both hydrolysable and nonhydrolysable nucleotide cofactors.

Importantly, Dmc1 displays a low DNA binding affinity in the absence of a nucleotide cofactor and Mei5–Sae3 did not change the affinity (Fig. 3F). Therefore, Mei5–Sae3 enhances Dmc1’s affinity for DNA in a manner that depends on the presence of a nucleotide co-factor, but not on an active hydrolytic cycle.

We also carried out the same analysis of the impact of Mei5–Sae3 on Dmc1–E157D in the presence of ATP. Without Mei5–Sae3, the mutant protein showed greatly reduced DNA-binding activity (*K*_D _= 2.2 µM) (Fig. 3D). Addition of Mei5–Sae3 to binding reactions had no impact. Finally, we substituted *E. coli* RecA protein for Dmc1 and showed that Mei5–Sae3 had no impact on the apparent *K*_D_ of RecA and DNA (Fig. 3E). Thus, the impact of Mei5–Sae3 on Dmc1 binding curves is species-specific.

We also compared the effect of Ca^2+^ on wild-type Dmc1’s DNA binding activity. We found that 250 µM Ca^2+^ supported a higher level of DNA binding activity as compared to 70 µM Ca^2+^ without Mei5–Sae3, and a similar level to that observed with 70 µM Ca^2+^ and Mei5–Sae3 (Supplementary Fig. S4A). Increasing the Ca^2+^ concentration to 1 mM further increased DNA binding; Dmc1–WT binding affinity at 1 mM was 8-fold higher than that observed in the absence of Ca^2+^ (Supplementary Fig. S4B).

### Analysis of Dmc1 filament length and allostery shows Mei5–Sae3, the E157D mutation, and high Ca^2+^ concentrations stabilize the active filament form and increase average Dmc1 filament lengths

**Figure 4.**
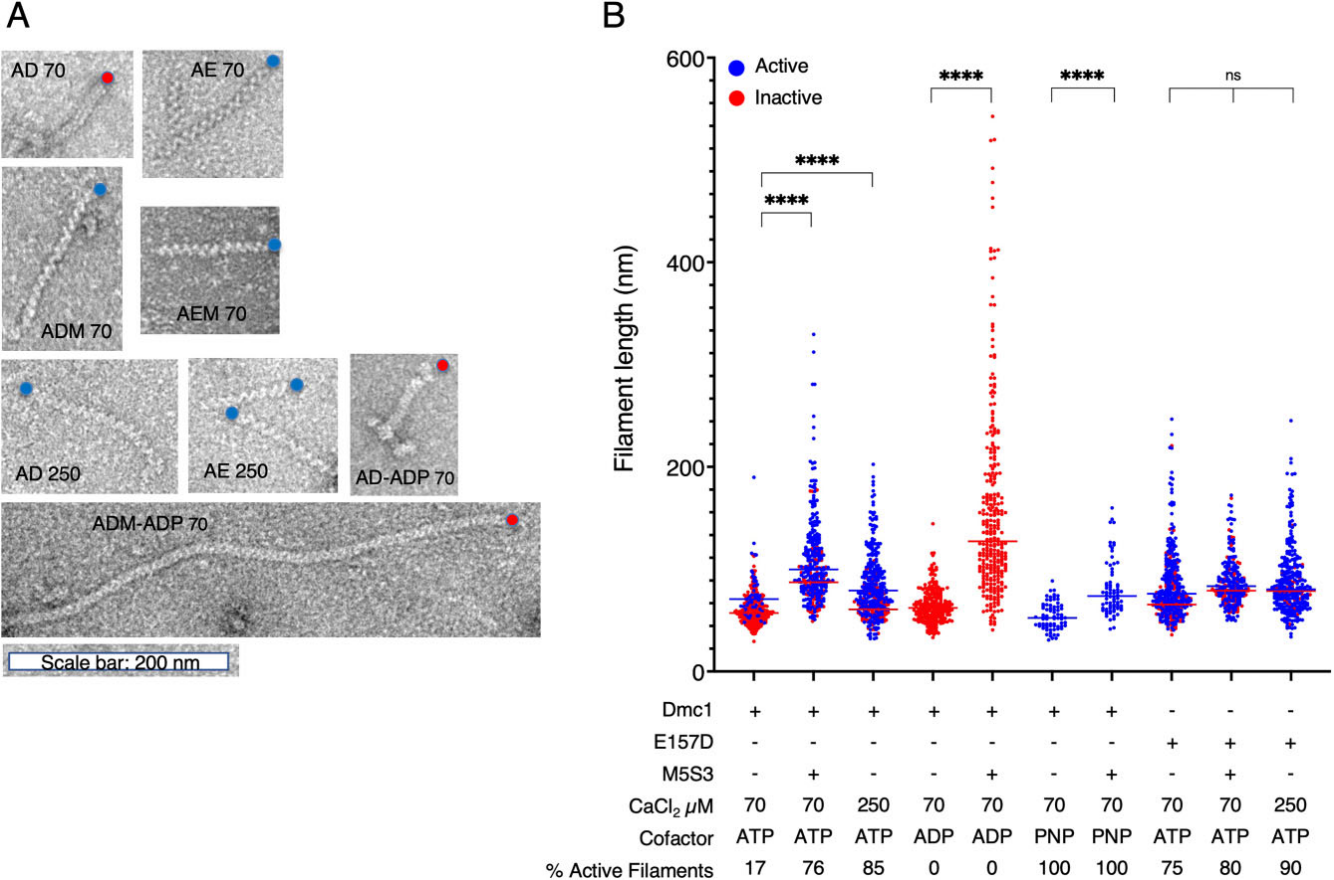
EM analysis of Dmc1 filaments shows Mei5–Sae3, high Ca^2+^, and the E157D mutation stabilize the extended form of the Dmc1 filament and increase filament lengths. (**A**) Micrographs of representative filaments. Filaments were assembled at the Ca^2+^ concentration indicated (μM). The protein acronyms are: A = RPA, D = Dmc1–WT, E = Dmc1–E157D, and M = Mei5–Sae3. Nucleotide cofactor AMP–PNP is abbreviated PNP. The representative filament shown for AD70 is in the compressed/inactive conformation with striations separated by ∼8 nm (marked with red dots). Inactive filaments are also shown when ADP is used in place of ATP. All other examples shown are active filaments with striations spaced ∼10 nm apart (marked with blue dots). (**B**) Scatter plot of individual filament lengths and their allosteric form as determined by the spacing of the filament’s striations. Inactive filaments are shown as red dots and active filaments are shown as blue dots. At 70 µM Ca^2+^ with ATP, the addition of Mei5–Sae3 (M5S3) to reactions with Dmc1–WT increased the number of filaments in the active form and the average filament length. When ADP replaced ATP, only compressed filaments formed. Surprisingly, Mei5–Sae3 caused a greater increase in average filament length with ADP than with ATP. For all statistical comparisons between samples, a two-tailed unpaired *t*-test was used with Welch’s correction to determine significance. ns = not significant, **** = significant at *P* < 0.0001.

We next examined the filaments formed by Dmc1–WT and Dmc1–E157D using negative staining and EM. This analysis allowed us to determine the lengths of individual filaments as well as distinguish between the two different allosteric forms based on the staining patterns along the filament contours. Representative fields from all EM experiments are shown in Supplementary Fig. S5. The staining method detects a filament form having striations spaced 10 nm apart. This is the active and extended filament form. The staining method also detects a different class of filaments with much less defined substructure. In some cases, filaments in this class have a barely discernable pattern with repeating substructures spaced at 8 nm intervals along filament contours. In other cases, no repeating pattern is discerned. Examples of this class of filament are shown in Fig. 4A, panels AD 70, AD-ADP 70, and ADM-ADP 70. We interpret these structures to be equivalent to the previously described inactive compressed filament form seen in the presence of ADP [19, 63] because they are the only forms we observe in the presence of ADP.

Using the same protein preparations, we noted that addition of the ssDNA-binding protein RPA increased the average Dmc1 filament length (compare Supplementary Fig S5A and B) as observed previously for Rad51 [64]. This is likely to result from blocks to Dmc1 filament elongation caused by ssDNA secondary structure given that ssDNA-binding proteins are well known to eliminate such structures without blocking recombinase filament elongation [65]. The structures formed by RPA alone are narrower than those formed by Dmc1 and have a “beads on a string” appearance (Supplementary Fig. S5L) [66], allowing segments of RPA filaments to be excluded from the analysis of Dmc1 filaments.

We examined filaments formed by Dmc1 on a 2627-nt ssDNA substrate in the presence of RPA, Mg^2+^, ATP, and 70 µM Ca^2+^ (Fig. 4A and B). We found that 83% of the Dmc1 filaments displayed the staining pattern consistent with the compressed form and only 17% were in the extended form. The average filament length was 66 ± 20 nm. Interestingly, in all experiments reported here, the filaments displayed uniform allostery, i.e. filaments had either extended or compressed appearance along their entire length. This uniformity suggested a high degree of allosteric cooperativity, a property previously described for allosteric behavior of Dmc1’s relative RecA [67]. Note that none of the Dmc1 filaments observed had contour lengths corresponding to complete coverage of the 2627-nt substrate, presumably because RPA was present in the binding reactions. RPA was used to prevent complications that might result from blocks to filament elongation by ssDNA secondary structure. We next examined the filaments under the same conditions but in the presence of Mei5–Sae3. Strikingly, with the addition of 2 µM Mei5–Sae3, 76% of Dmc1 filaments were in the extended form, 4.5-fold higher than in the control reaction without Mei5–Sae3 (Fig. 4B). In addition, the average filament length was 96 ± 36 nm, 1.5-fold longer than in the control. These results indicate that Mei5–Sae3 acts as an allosteric effector of Dmc1, stabilizing the active/extended filament form.

We also tested the ability of Dmc1 to form filaments in the presence of ADP and AMP–PNP. As expected, all filaments formed in the presence of ADP displayed the compressed filament form, regardless of whether Mei5–Sae3 was included in the reactions or not (Fig. 4 and Supplementary Fig. S5I  and J). Notably, addition of Mei5–Sae3 to ADP containing reactions caused a dramatic 2.3-fold increase in average filament length from 64 ± 17 nm to 158 ± 94 nm. These results have two major implications. First, the ability of Mei5–Sae3 to stabilize the active filament form of Dmc1 depends on the presence of ATP. Second, Mei5–Sae3’s influence on Dmc1 filament length is not restricted to reactions containing ATP; Mei5–Sae3 has an even greater impact on Dmc1 filament length in the presence of ADP than in the presence of ATP. The ability of Mei5–Sae3 to increase Dmc1 filament length in the presence of ADP is consistent with its ability to enhance Dmc1’s DNA-binding affinity (Fig. 3B). Using AMP–PNP yielded almost exclusively active filaments and addition of Mei5–Sae3 caused a significant, 1.5-fold increase in average filament length (Fig. 4B). Thus, polarization and EM analysis show that Mei5–Sae3 can enhance Dmc1’s DNA-binding activity using ATP, ADP or AMP–PNP as cofactors.

Importantly, we did not observe significant Dmc1 filament formation under equivalent conditions that lacked nucleotide cofactor, regardless of the presence of Mei5–Sae3 (Supplementary Fig. S5K).

Further experiments will be required to determine why Mei5–Sae3 promotes formation of longer Dmc1 filaments with ADP as compared to ATP. One possibility is the elongation involving the inactive filament form is more energetically favorable than that involving the active form because it does not distort the DNA to the same extent. Another possibility is that ATP hydrolysis at filament ends increases the rate of protomer dissociation relative to that when no hydrolysis is occurring. Finally, we examined filament formation by Dmc1–E157D. Consistent with the ability of this mutant to bypass Mei5–Sae3 function *in vivo*, we found that 75% of the filaments that form with 70 µM Ca^2+^ in the presence of ATP display the extended conformation (Fig. 4B). Thus, the fraction of Dmc1–E157D filaments displaying the extended conformation in the absence of Mei5–Sae3 is 4.4-fold higher for Dmc1–E157D than for Dmc1–WT. The average filament length was 83 ± 33 nm, intermediate to the average filament length seen for Dmc1–WT without Mei5–Sae3 and for Dmc1–WT with Mei5–Sae3. Addition of Mei5–Sae3 did not have a significant impact on Dmc1–E157D filament conformation or length (Fig. 4B; Supplementary Fig. S5F  and G). Thus, the E157D mutation mimics the impact of Mei5–Sae3 on filament allostery, as does increasing Ca^2+^ concentration.

### Mei5–Sae3 can stabilize the active form of Dmc1 filaments without blocking the ATP hydrolytic cycle

The ability of Mei5–Sae3 to stabilize the active form of the Dmc1 filaments in the presence of ATP suggested that the underlying molecular mechanism involves stabilization of the ATP bound protomer form within Dmc1 filaments. One mechanism through which Mei5–Sae3 could stabilize the ATP-bound protomer form is by blocking ATP hydrolysis. Yet previous studies using the fission yeast and mouse Mei5–Sae3 homologs showed that they cause their cognate recombinases to hydrolyze more ATP than occurs in control reactions [47, 48, 50].

**Figure 5.**
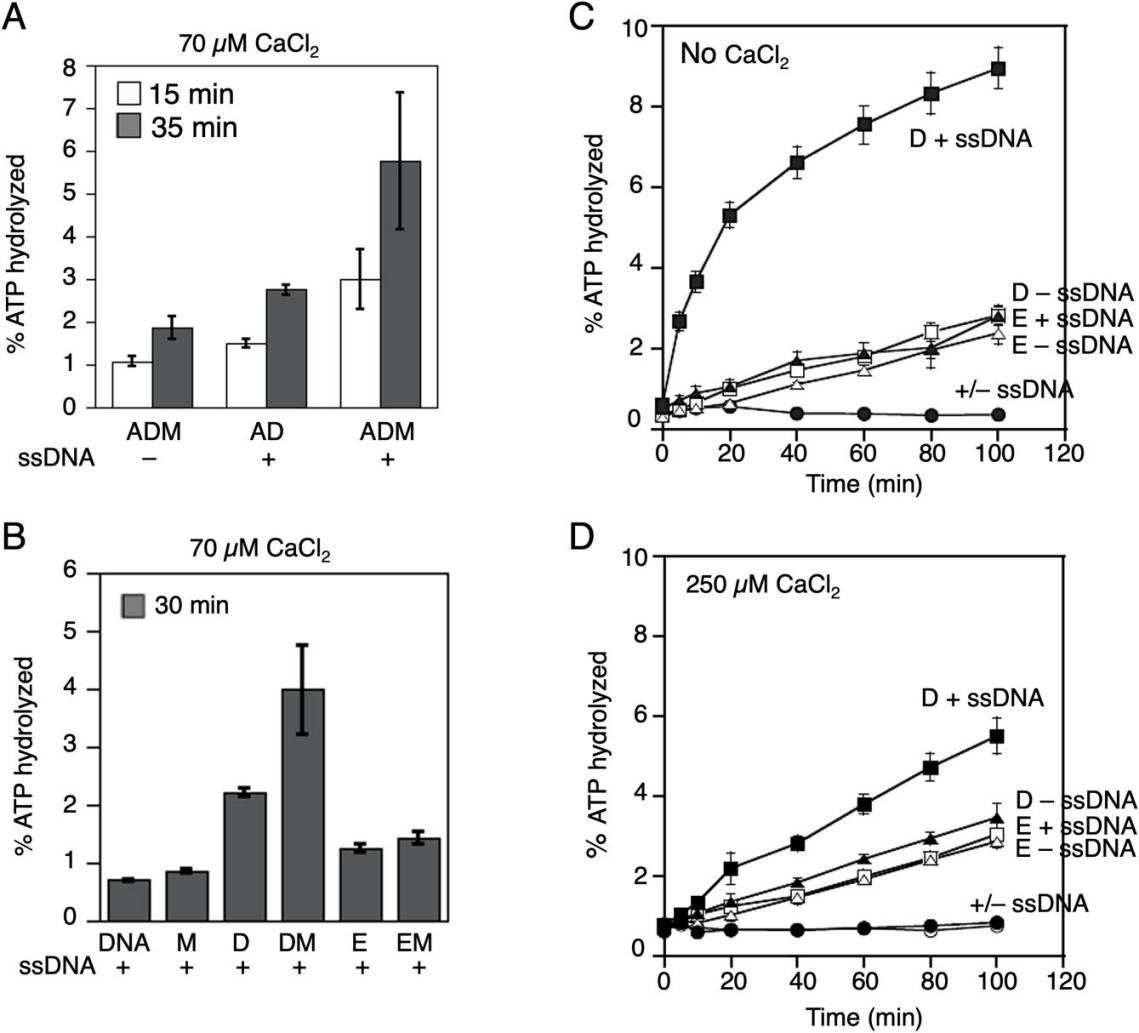
Mei5–Sae3 stimulates Dmc1’s ATPase activity. ATP hydrolysis was monitored by TLC. (**A** and **B**) Mei5–Sae3 stimulates the ssDNA-dependent ATP hydrolysis of Dmc1–WT, but not of Dmc1–E157D. This Mei5–Sae3 stimulatory effect is largely ssDNA-dependent. (**C**) Dmc1–E157D lacks DNA-dependent ATPase activity, even in the absence of Ca^2+^, which inhibits ATP hydrolysis. (**D**) With 250 µM Ca^2+^, Dmc1–WT still has ATPase activity but Dmc1–E157D does not. The protein acronyms and concentrations are: A = RPA (0.2 µM), D = Dmc1 (3 µM), E = Dmc1–E157D (3 µM), M = Mei5–Sae3 (2 µM). The % ATP hydrolyzed at the indicated time are plotted as averages ± SEM (*n* = 3). Note that although Dmc1–E157D displays a strong defect in DNA binding under some conditions, e.g. the experiment in Fig. 3D, the conditions used for this experiment support a similar level of DNA binding for Dmc1 and Dmc1–E157D see Supplementary Fig. S8.

To better understand the mechanism through which Mei5–Sae3 stabilizes the active Dmc1 filaments, we first used a conventional TLC assay to measure Dmc1’s ATPase activity in the presence and absence of Mei5–Sae3 (Fig. 5 and Supplementary Fig. S6). We found that addition of Mei5–Sae3 enhanced the amount of ATP hydrolyzed by Dmc1 ~2-fold in reactions with 70 µM Ca^2+^ (Fig. 5A). We also assayed the ATPase activity of Dmc1–E157D, which was designed to emulate the ATPase deficient form of bacterial recA protein recA-E96D [33, 34]. We did not detect any DNA-dependent ATPase activity for the mutant protein as predicted, and the addition of Mei5–Sae3 did not alter this result (Fig. 5B). Additional attempts to detect ATPase activity for Dmc1–E157D were carried out under two additional conditions: with no Ca^2+^ present (Fig. 5C) to prevent any Ca^2+^-dependent inhibition of ATPase, or at 250 µM Ca^2+^ (Fig. 5D), which supports similar levels of D-loop formation for both the wild-type and mutant recombinases. We did not detect any DNA-dependent ATPase activity under either condition, as expected. Given that the small amount of DNA-independent ATPase activity was nearly identical for Dmc1–WT and Dmc1–E157D, we presume that it the result of a small amount of a contaminating ATPase. This is a common problem when studying eukaryotic RecA family members because of the very low level of their intrinsic ATPase activity e.g. [37]. The opposing effects of Mei5–Sae3 and the E157D mutation on Dmc1’s ability to hydrolyze ATP indicate that Mei5–Sae3 and E157D stabilize the extended form of Dmc1 filaments by different mechanisms.

**Figure 6.**
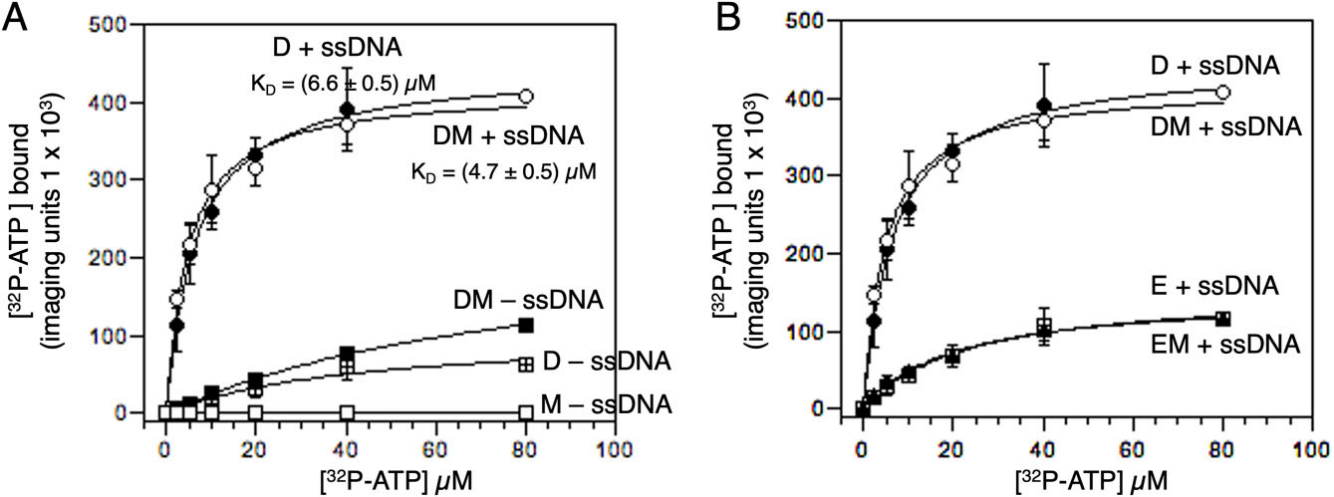
Mei5–Sae3 does not enhance ATP binding to Dmc1–WT or Dmc1–E157D. (**A**) When Dmc1–WT (abbreviated D) was added last to the reaction, Dmc1 binds ATP efficiently with or without Mei5–Sae3 (abbreviated M). Without ssDNA, Dmc1 or Mei5–Sae3 binds ATP at low to background level. (**B**) Dmc1–E157D (abbreviated E) binds less ATP than Dmc1–WT and Mei5–Sae3 has no effect on its binding. Data are plotted as averages ± SEM (*n* ≥ 3). Note that the data for D and DM + are repeated in both panels to facilitate comparison with other conditions.

We next measured the ability of Dmc1–WT to bind ATP in the presence and absence of Mei5–Sae3 using a filter binding assay (Fig. 6 and Supplementary Fig. S7). In establishing this assay, we showed that Mei5–Sae3 alone does not bind ATP (Fig. 6A), consistent with previous findings for Mei5–Sae3 homologs [48, 68]. The result showed that Dmc1–WT binds ATP with the same apparent affinity in the presence and absence of Mei5–Sae3 (*K*_D_ ∼5–6 µM). Additionally, we find that Dmc1’s ATP-binding activity is strongly DNA-dependent, both in the presence and absence of Mei5–Sae3. Considering the results of the DNA binding experiments described above, which confirm that Dmc1 requires ATP for its DNA-binding activity, the results indicate that Dmc1 ATP and DNA binding are mutually interdependent, similar to previous results using human Dmc1 and fission yeast Rad51 [50, 69].

We also measured the ATP binding activity of Dmc1–E157D and found it was significantly reduced, saturating at a level 4-fold lower than that of the wild-type protein (Fig. 6B). Thus, the ATP-binding defect conferred by the Dmc1–E157D mutation may contribute to the protein’s lack of ATPase activity. This is interesting considering the strong impact of the mutation in stabilizing the ATP-dependent active filament form as shown in Fig. 4. Addition of Mei5–Sae3 had no effect on the ATP-binding curve of Dmc1–E157D consistent with other results.

During these experiments, we found that the order of addition of reaction components had a drastic impact on Dmc1’s ATP and DNA-binding activities unless Mei5–Sae3 was present in reaction mixtures. In the absence of Mei5–Sae3, when Dmc1 was added to reaction mixtures before ATP, the maximum amount of ATP binding observed at saturation was 4- to 6-fold less than that observed when ATP was present at the time of Dmc1 addition (Supplementary Fig. S7A–C). When Mei5–Sae3 was present and nucleotide cofactor was added after Dmc1 (Supplementary Fig. S7B), the plateau level of ATP binding observed were like those observed when Dmc1 was added to reactions after addition of nucleotide cofactor (Supplementary Fig. S7A), indicating that Mei5–Sae3 promotes retention of Dmc1’s nucleotide-binding activity. Consistent with the interdependence of ATP and DNA binding described above, we also saw a dramatic reduction in ATP binding when DNA was added to reactions after Dmc1 (Supplementary Fig. S7C). In contrast to adding ATP last, Mei5–Sae3 had a much smaller impact on Dmc1’s ATP-binding activity when DNA was added last. These data suggest that the ATP and DNA-binding activities of Dmc1 in our preparations tend to be lost unless ATP and DNA are present in reaction mixtures at the time of Dmc1 addition. Mei5–Sae3 can partially rescue wild-type Dmc1’s ATP binding, again suggesting that it stabilizes an ATP- and DNA-binding-competent conformation of Dmc1. The *in vivo* significance of this finding is unclear given the general availability of ATP.

**Figure 7.**
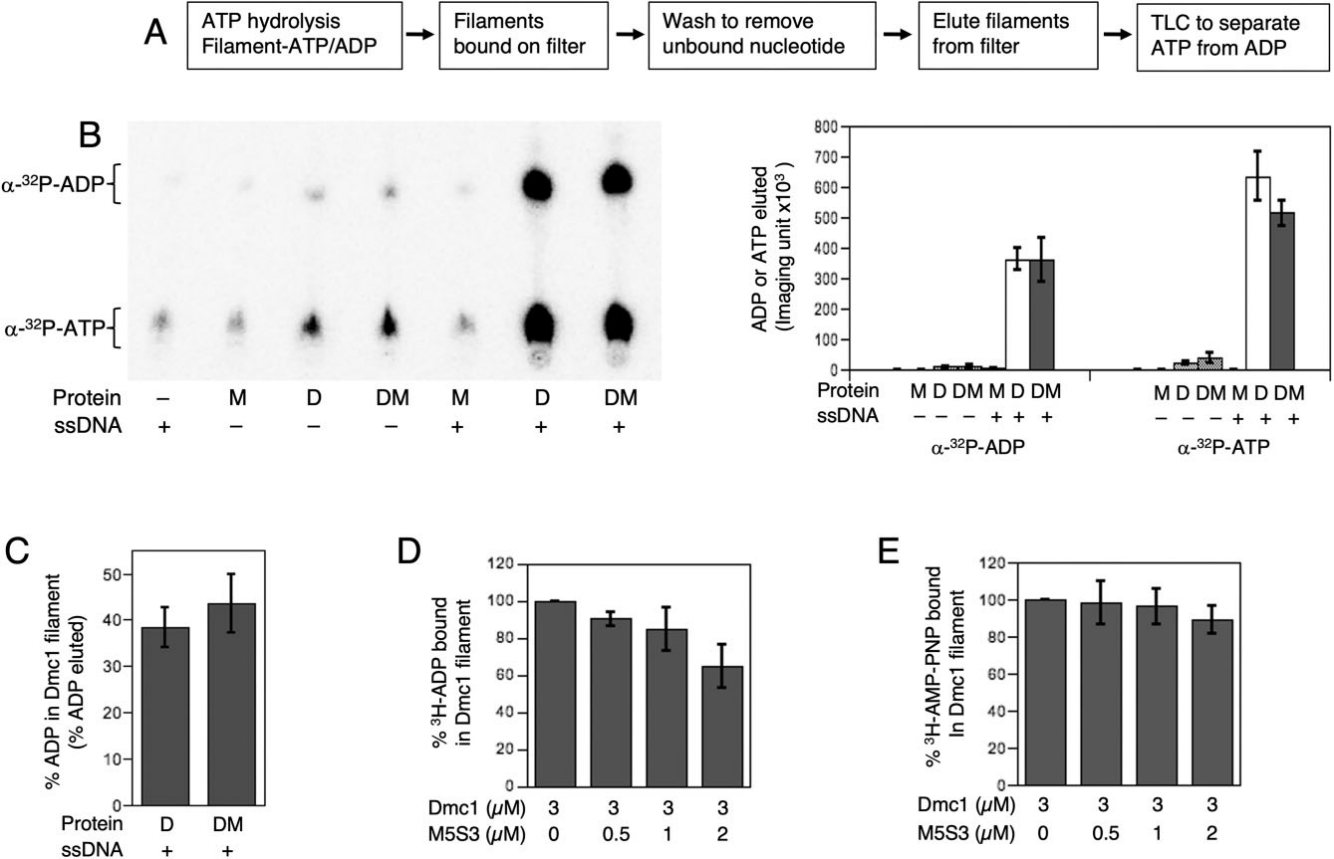
Dmc1 filament profiling and the impact of Mei5–Sae3 on nucleotide cofactor binding. (**A**) Scheme illustrating the profiling by nitrocellulose filter binding, elution, and TLC, which monitors radioactive ADP and ATP bound in the Dmc1 filaments. Dmc1 was added last to the reactions to initiate ATP hydrolysis. A representative TLC radiogram is shown. The protein concentrations and acronyms are: D = Dmc1–WT (3 µM), M = Mei5- Sae3 (1 µM). (**B**) The amount of radioactive ADP and ATP eluted from nitrocellulose filter was quantified from the TLC radiograms. (**C**) The % ADP bound in Dmc1 filaments is derived from the ratio of ADP/(ADP + ATP). (**D**) Mei5–Sae3 facilitates ^3^H-ADP release from Dmc1 filaments pre-bound with ^3^H-ADP. (**E**) Mei5–Sae3 has little or no effect in releasing ^3^H-AMP–PNP from Dmc1-filaments pre-bound with the same amount of nucleotide. The data are plotted as averages ± SEM (*n* ≥ 3). The data are derived and quantified from TLC radiograms and plotted as averages ± SEM (*n* = 3).

We further assessed the impact of Mei5–Sae3 on Dmc1’s hydrolytic cycle by profiling the nucleotide species bound to Dmc1 filaments in the presence of and absence of Mei5–Sae3 (Fig. 7). We first used the same filter binding method described above for measurement of ATP binding to trap protein-bound nucleotide. Unbound nucleotide was washed away, and bound nucleotide eluted from filters using detergent and proteinase treatment (Fig. 7A). We then determined the yields of eluted radiolabeled ATP and ADP following their separation via TLC. Using this method, we detected filament bound ATP and ADP from reactions involving Dmc1–WT at 70 µM Ca^2+^ (Fig. 7B). The ratio of ATP to ADP in the eluted sample was 1.8. This finding contrasts with prior results using human Dmc1 [23] and indicates that ADP accumulates to a significant level following hydrolysis rather than being quickly released, similar to human Rad51 [22, 29]. Addition of Mei5–Sae3 under the same reaction conditions resulted in a minor (20%) decrease in the amount of ATP bound without a change in the level of ADP (Fig. 7B and C). These results are consistent with the acceleration of ATP hydrolysis seen upon Mei5–Sae3 addition if ADP dissociation is the rate limiting step in the hydrolytic cycle without Mei5–Sae3 but ATP binding is limiting with Mei5–Sae3.

A previous report on the mouse Swi5–Sfr1 identified conditions under which it enhances ADP release by RAD51 [48]. Thus, we carried out a similar analysis with budding yeast Mei5–Sae3 and Dmc1–WT (Fig. 7D). In this experiment, 3 µM Dmc1 was incubated with an equimolar amount of ADP-3H. Varying amounts of Mei5–Sae3 were then added and all unbound ADP washed away. This analysis showed that increasing amounts of ADP were released as the concentration of Mei5–Sae3 increased (Fig. 7D). At 2 µM Mei5–Sae3, only 65% of the ADP that was originally bound was retained. In contrast to the results with ADP, a similar analysis using AMP–PNP showed little or no AMP–PNP was displaced by Mei5–Sae3 (Fig. 7E). Given that we could detect a similar activity of Mei5–Sae3 on ADP release as previously demonstrated for mouse Swi5-Sfr1, these findings suggest that ADP release in the absence of accessory proteins is rate limiting across the eukaryotic RecA homologs.

Given our novel finding that Dmc1’s nucleotide-binding activity is interdependent with DNA binding, we considered the possibility that the enhancement of Dmc1’s ATP hydrolytic activity by Mei5–Sae3 is an indirect consequence of increased DNA binding, as shown in Fig. 3. To address this concern, we repeated the DNA binding analysis using the same DNA concentration as that used for the ATPase assays (6 µM nucleotide rather than 0.25 µM, the concentration used in the experiments shown in Fig. 3 and Supplementary Fig. S3). We note that curves from both the Mei5–Sae3 containing reactions and the control reactions approach saturation at 3 µM Dmc1, the concentration used in the ATPase assays. The modest (10%) difference in FP signal at 3 µM Dmc1 is unlikely to account for the 2-fold increase in Dmc1’s ATPase activity supported by Mei5–Sae3. A second line of evidence that supports the view that Mei5–Sae3 increases the rate of hydrolysis of ATP by DNA-bound Dmc1 protomers is provided by ATP-binding data. Given that ATP binding depends on DNA binding, the level of ATP binding provides an indirect measure of the amount of DNA binding. As mentioned above, addition of Mei5–Sae3 did not significantly alter ATP binding; implying that DNA binding is not significantly increased by Mei5–Sae3 under the assay conditions. Therefore, under these conditions, enhancement of ATP hydrolysis by Mei5–Sae3 is not an indirect consequence of enhanced DNA binding.

## Discussion

Here, we show that Mei5–Sae3 displays a greater impact on Dmc1’s activity when assayed at a physiologically relevant Ca^2+^ concentration as compared to the high Ca^2+^ concentrations used by us and others in prior studies. We previously showed that although Dmc1 requires Ca^2+^ ions to display robust D- loop activity, the concentration of Ca^2+^ required for such activity is reduced to physiological levels if reactions also contain Mg^2+^ ions at physiological levels [59]. The use of physiological conditions appears to be critical to reliable characterization of Mei5–Sae3 function given that the suppression of ATPase activity that occurs at higher Ca^2+^ concentrations mimics the functional impact of Mei5–Sae3 on Dmc1 allostery and DNA-binding activity.

### Potential function of Mei5–Sae3 in influencing Dmc1’s DNA-binding activity

To function, recombinases must assemble specifically on relatively rare tracts of ssDNA. This requirement is complicated by the fact that the reaction mechanism that allows recombinase filaments to find homologous target sites involves formation of hybrid dsDNA, a transition driven by product stability rather than by harnessing the energy from ATP hydrolysis [17]. Consequently, recombinases have considerable intrinsic dsDNA-binding activity. This is especially true for the eukaryotic recombinases Rad51 and Dmc1. Because of this intrinsic dsDNA binding activity, and the fact that the quantity of dsDNA in the cell vastly exceeds that of ssDNA, mechanisms that confer ssDNA binding specificity to recombinases *in vivo* are required to overcome potentially detrimental consequences of off-pathway dsDNA binding. We speculate that regulation of Dmc1 filament formation and stability by Mei5–Sae3 contributes to the binding specificity of Dmc1 for ssDNA. Direct protein–protein interactions between RPA, Mei5–Sae3, and Dmc1 [53] may help limit Mei5–Sae3’s enhancement of Dmc1’s DNA-binding activity to ssDNA *in vivo*. Consistent with this possibility, Dmc1–E157D, which has lost regulation by Mei5–Sae3, displays very high levels of off-pathway dsDNA binding *in vivo* [51]. Furthermore, the off-pathway Dmc1 complexes that accumulate in translocase-defective cells do not depend on Mei5–Sae3, indicating that Mei5–Sae3’s role in enhancing Dmc1 focus formation is normally specific for ssDNA *in vivo*.

Another possible function for regulation of Dmc1 filaments by Mei5–Sae3 is to provide a mechanism to activate or inactivate Dmc1’s homology search and strand exchange activities of filaments after their formation. There could be utility in keeping Dmc1 filaments inactive during early stages of meiotic prophase as chromosome structure is remodeled, or in inactivating Dmc1’s activity at later stages of prophase to allow Dmc1’s homolog Rad51 to “take over” residual DNA break repair at late stages [58, 70–72]. In this context, we note that the ability of Mei5–Sae3 to stabilize the ATP-bound active filament form is likely to be the physiologically relevant activity given the relative abundance of ATP and ADP in the cell. It is not yet clear if the ability of Mei5–Sae3 to stabilize the inactive filament form in the presence of ADP reflects a biologically relevant process or not.

**Figure 8.**
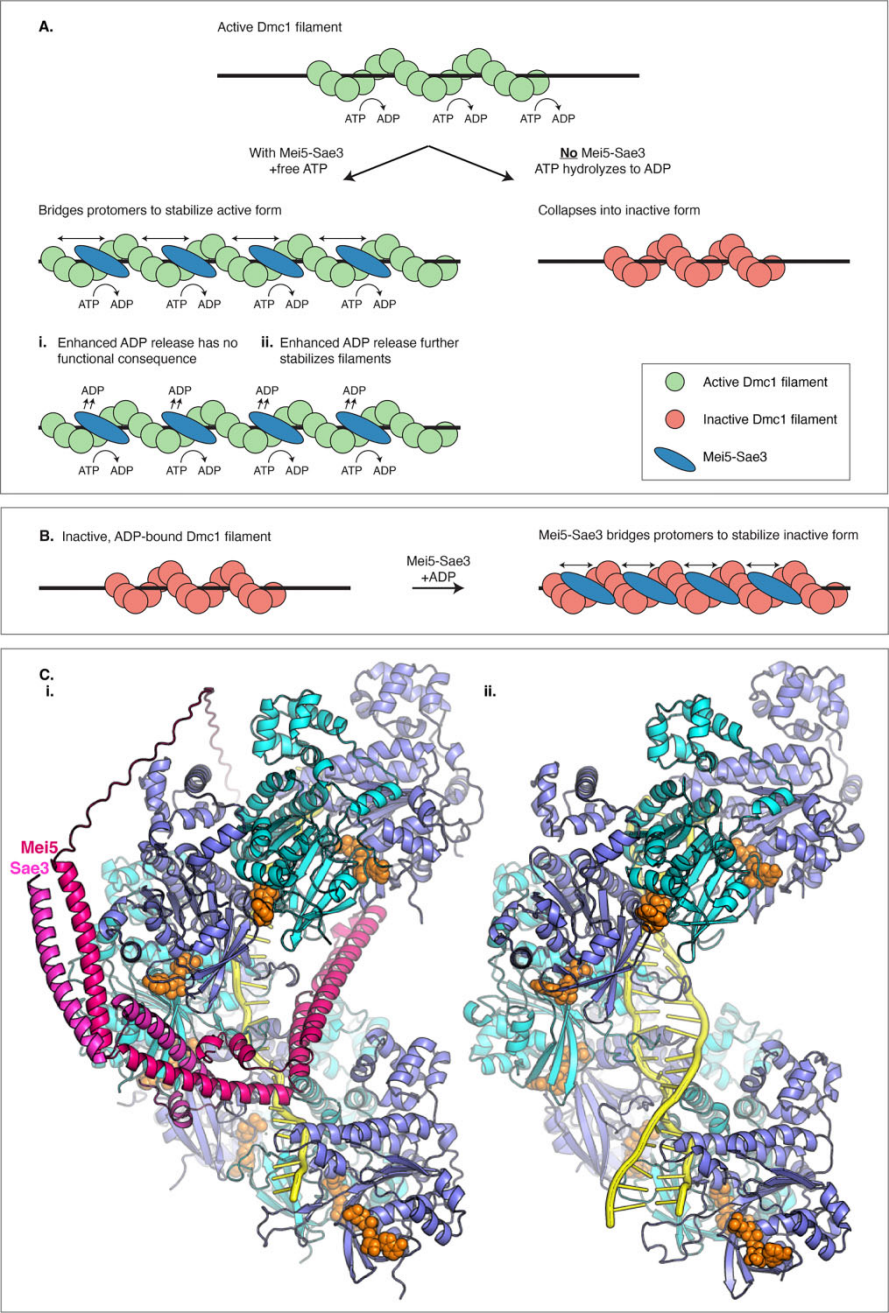
Models for the mechanism of Mei5–Sae3 stimulation of Dmc1’s DNA binding activity. (**A**) In the absence of Mei5–Sae3, ATP hydrolysis results in the conversion of extended active filaments into the collapsed, inactive form. Model (i) protomer bridging by Mei5–Sae3 stabilizes the active filament form. Mei5–Sae3 also enhances ADP release, but that activity does not contribute to Dmc1’s DNA-binding activity. Model (ii) protomer bridging stabilizes the active filament form and enhances ADP release. Enhanced ADP release further enhances Dmc1’s DNA binding activity. (**B**) Mei5–Sae3 stabilizes inactive, ADP-bound filaments in the collapsed inactive filament form via protomer bridging providing evidence that filament stabilization does not require alteration of the hydrolytic cycle. (**C**) Alphafold3 modeling of Mei5–Sae3 supports the protomer-bridging model for Dmc1 filament stabilization. Mei5–Sae3 is predicted to occlude the second strand of duplex DNA. (i) Alphafold3 model of 7 Dmc1 protomers (alternating shades of blue) with ssDNA (yellow), ATP (orange spheres), and one Mei5–Sae3 heterodimer (shades of magenta). (ii) An experimentally determined structure of Dmc1 bound to ATP and duplex DNA, shown in the same orientation (PDB ID: 7ej7) [73].

Our work provides *in vitro* evidence that Mei5–Sae3 has at least two partially separable effects on Dmc1 filaments (Fig. 8A). First, Mei5–Sae3 enhances Dmc1 DNA-binding affinity (Fig. 3) and filament length (Fig. 4) irrespective of the nucleotide cofactor (ATP, ADP, and AMP–PNP). Importantly, although this enhancement of Dmc1 filament formation/stability is observed in the presence of ATP, ADP, and AMP–PNP, it is not observed in the absence of nucleotide (Fig. 3E and Supplementary Fig. S5K). These results indicate that Mei5–Sae3 promotes Dmc1 filament formation, stability, and/or elongation provided that Dmc1 is competent to bind DNA; competency is dependent on a nucleotide cofactor. The findings with AMP–PNP and ADP are particularly significant because they indicate that Mei5–Sae3 can enhance Dmc1 filament formation/length independently of its impact on the ATP hydrolytic cycle. Though Iwasaki *et al.* previously showed that fission yeast Swi5–Sfr1 allows Rad51 to displace RPA from ssDNA with either ATP or AMP–PNP as a cofactor [50], this is the first time to our knowledge that an ADP-dependent effect has been observed for any family member and the first time the AMP–PNP effect has been demonstrated for Mei5–Sae3.

Notably, whereas an effect of fission yeast Swi5–Sfr1 on Rad51 can be observed with AMP–PNP [50], and we similarly see an effect of AMP–PNP with budding yeast Mei5–Sae3 on Dmc1, across the Swi5–Sfr1 family of proteins, the stimulatory effect of Ca^2+^ and Swi5–Sfr1 are mutually exclusive (Fig. 1) [49, 61, 74]. Similarly, Ca^2+^ and nonhydrolysable ATP analogs were found to have separable effects on RecA and RecA homolog filaments and strand exchange *in vitro* [23, 26, 75]. Collectively, these results indicate that Ca^2+^ affects RecA homolog filaments beyond preventing ATP hydrolysis. Though more work is required to understand subtle differences as to the differential stimulatory effects of Ca^2+^, AMP–PNP, and Swi5–Sfr1 family proteins on Rad51’s and Dmc1’s activities across species, it is worth noting that a key difference between human Rad51 and Dmc1 is that the former retains ADP whereas the latter does not [22, 23]. While not novel to our study, this result is worth emphasizing in light of numerous biochemical reconstitution studies that rely on high levels of Ca^2+^ to achieve Rad51/Dmc1 D-loop formation. Here, we demonstrate that high Ca^2+^ masks the effect of Mei5–Sae3 *in vitro*, although Dmc1 focus formation and D-loop formation is totally dependent on this effector *in vivo*.

In spite of our finding that Mei5–Sae3 can stabilize Dmc1 filaments independently of the ATP hydrolytic cycle, we also find that it enhances ADP release and accelerates the hydrolytic cycle, as shown previously in other systems (Figs 5 and 7) [48]. Importantly, ATP supports significantly tighter DNA binding than ADP or AMP–PNP (Fig. 3). Furthermore, the enhanced rate of ATP hydrolysis is not caused by increased ATP binding (Fig. 6) nor is it associated with increased DNA binding (Fig. 3A at 3000 nM Dmc1), consistent with ATP hydrolysis playing a role in filament stabilization. On the other hand, ATP might be more effective in promoting filament stability than AMP–PNP because AMP–PNP does not provide a perfect structural mimic of ATP, as is the case for *E. coli* RecA [76]. These results led us to consider two models to explain why Mei5–Sae3 has significant activity with AMP–PNP, but a higher level of activity with ATP (Fig. 8A). First, in the case that AMP–PNP does not perfectly substitute for the function of ATP prior to hydrolysis, all enhancement of filament formation by Mei5–Sae3 could result from filament bridging with the impact of Mei5–Sae3 on the hydrolytic cycle being secondary and inconsequential with respect to Dmc1’s DNA-binding activity (Fig. 8Ai). Alternatively, if the only significant functional difference between AMP–PNP and ATP is the absence of hydrolysis, Mei5–Sae3’s protomer bridging may provide only partial enhancement of Dmc1’s DNA-binding activity with the associated acceleration of ATP hydrolysis being required for the full level of enhancement (Fig. 8Aii). Further studies will be required to distinguish these models. We favor the first model because of its simplicity and because filament profiling showed that Mei5–Sae3 reduces rather than enhances the amount of filament-bound ATP at steady state (Fig. 7). Dmc1 protomer bridging alone by Mei5–Sae3 explains the enhanced stability of Dmc1 filaments in the absence of the ATP hydrolytic cycle (Fig. 8B).

How do members of the Swi5–Sfr1 family exert their effect on their cognate recombinases? Based on the crystal structure of fission yeast Swi5–Sfr1, it has been suggested that the heterodimer may fit within the groove of Rad51/Dmc1 nucleoprotein filaments [68]. In this regard, structural modeling using AlphaFold3 [77] provides a moderately high confidence model (pTM = 0.7, ipTM = 0.67) in which a Mei5–Sae3 heterodimer bridges five consecutive Dmc1 protomers bound to ssDNA (Fig. 8C and Supplementary Fig. S9A). The predicted bridging interaction provides support for a model in which protomer bridging is responsible for enhanced Dmc1 binding. We note than Dmc1 protomer bridging by Mei5–Sae3 is also consistent with the ability of Mei5–Sae3 to stabilize clusters of three protomers on short DNA substrates by reducing off-rates [45]. Interestingly, the model also positions one of three alpha helical domains of Mei5–Sae3 such that it partially occludes the high affinity DNA-binding site of Dmc1 (Supplementary Fig. S9B). Although binding of the initial single DNA strand is still allowed, comparison to the experimental structure of duplex-bound Dmc1 shows that this helical segment of Mei5–Sae3 places a negatively charged protein surface where the negatively charged backbone of the 2nd DNA strand would lie. If the predicted strand occlusion is proven to be correct, the structure would provide a mechanistic explanation for *in vivo* observations suggesting Mei5–Sae3 confers ssDNA binding specificity to Dmc1 during filament assembly [5, 6, 51]. Experiments are required to test the model. Regardless of the stand occlusion feature of the model is correct or not, the AlphaFold3 model supports the proposal that Mei5–Sae3 bridges Dmc1 protomers [68].

### Meiotic recombination does not require ATP hydrolysis by Dmc1

The results presented here confirm our prediction that the E157D mutation blocks the ATPase activity of Dmc1 [51] and stabilizes the active filament form. They also confirm the prediction that the mutant protein would display a high level of D-loop activity. These predictions were based on the *in vivo* behavior of the *Dmc1–E157D* allele [17], and on prior biochemical characterization of the corresponding mutation in the *E. coli* RecA protein, RecA–E96D [16, 33]. Although these predictions were fulfilled, the purified protein displayed unexpected defects including dependency on high Ca^2+^ concentrations for D-loop activity, defective ATP and DNA binding, and temperature instability. Together these defects limited the utility of our preparations of mutant protein as a tool for testing the normal function of Mei5–Sae3. Particularly problematic is the high Ca^2+^ requirement for D-loop activity given that high Ca^2+^ blocks ATPase activity. Nonetheless, the experiments presented here support the view that Dmc1’s ability to promote HR is independent of its normal ATPase activity. This result is consistent with the results of previous studies of Dmc1 homologs RecA and Rad51 [15, 37, 78–82]. The results here also provide strong support for the view that the critical mechanism through which Mei5–Sae3 promotes Dmc1-mediated recombination is via stabilization of the active filament form. The biological relevance of unexpected ability of Mei5–Sae3 to stabilize the inactive filament form in the presence of ADP remains to be determined. We also acknowledge that the results presented here do not formally preclude the possibility that additional mechanisms contribute to the efficiency of meiotic recombination in *dmc1–E157D mei5* and *dmc1–E157D sae3* cells.

### Concluding remarks

Mei5–Sae3 stabilizes the active filament form of Dmc1 by a mechanism that does not require its ability to stimulate Dmc1’s ATP hydrolytic activity. The hydrolysis-independent Dmc1 stimulatory activity of Mei5–Sae3 is shown to also be active in the presence of ADP where it enhances inactive filament formation. These results along with modeling using AlphaFold3 support the hypothesis that Mei5–Sae3 stimulates the DNA-binding activity of Dmc1 by a mechanism involving protomer bridging.

## Supplementary Material

gkaf1085_Supplemental_File

## Data Availability

The data that support the findings of this study are presented in the main text paper and in the online Supplementary Information file. Further information and requests for resources and reagents should be directed to and will be fulfilled by the corresponding author Douglas K. Bishop (dbishop@uchicago.edu)
