# Supplementary material for: Mei5–Sae3 stabilizes both active and inactive forms of Dmc1 filaments independently of its impact on ATP hydrolysis
Source: Nucleic Acids Res. 2025 Nov 6;53(20):gkaf1085. doi: 10.1093/nar/gkaf1085 (PMC12596195; doi:10.1093/nar/gkaf1085)
Supplement: gkaf1085_Supplemental_File [file gkaf1085_supplemental_file.pdf]

SUPPLEMENTARY FIGURES S1-S8

MANUSCRIPT TITLE

Mei5-Sae3 stabilizes both active and inactive forms of Dmc1 filaments independently of its impact on ATP hydrolysis.

Yuen-Ling Chan[1]*, Diedre Reitz[2,3]*, Brian Budke[1], Phoebe A. Rice[4], and Douglas K. Bishop[1,2]†

1. Department of Radiation and Cellular Oncology, Department of Molecular Genetics and Cell Biology, University of Chicago, Chicago, Illinois, USA
2. Committee on Genetics, Genomics, and Systems Biology, University of Chicago, Chicago, Illinois, USA
3. Current Address: Department of Microbiology and Molecular Genetics, University of California, Davis, Davis, California, USA
4. Department of Biochemistry and Molecular Biology, University of Chicago, Chicago, Illinois, USA

*These authors made equal contributions to this study
†corresponding author: dbishop@uchicago.edu

Supplementary Figure S1

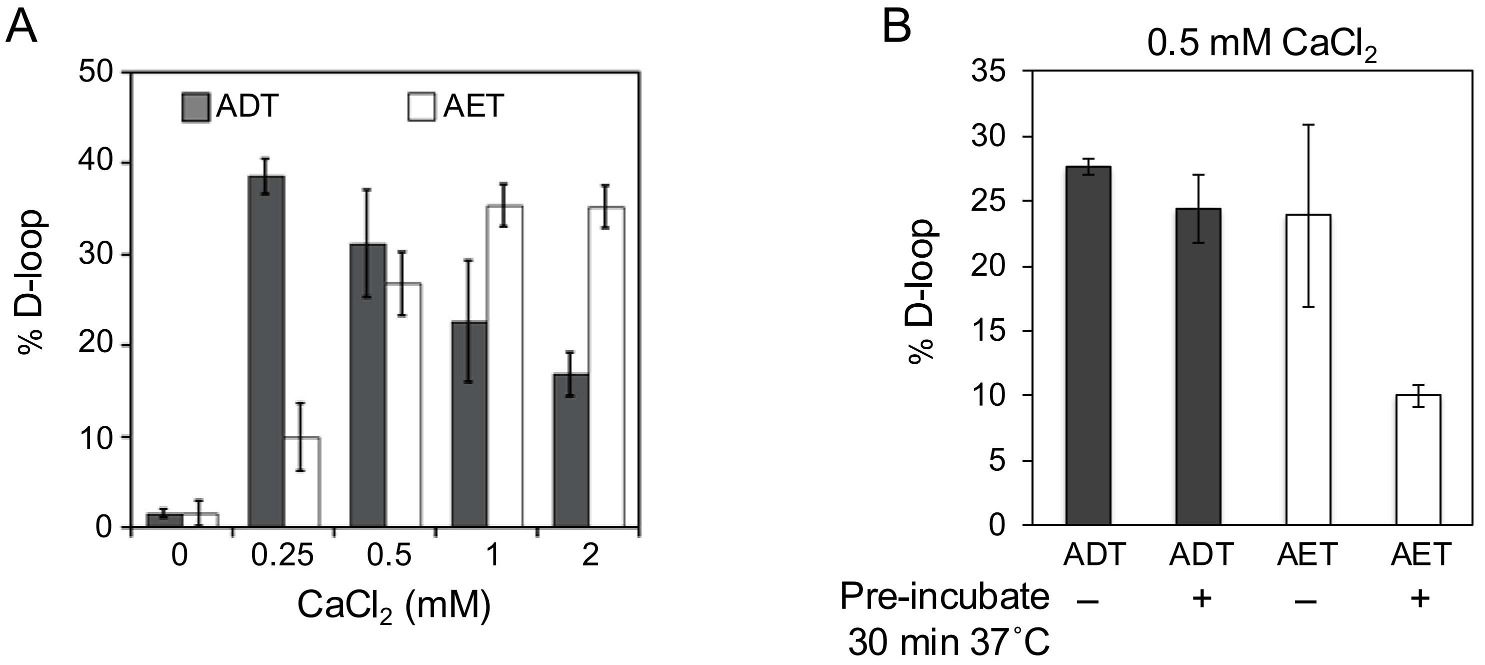

Supplementary Figure S1. Dmc1-E157D forms D-loops at high Ca^{2+} concentrations but is heat unstable. (A) Dmc1-WT forms more D-loops at low Ca^{2+} concentrations while Dmc1-E157D makes more D-loops at high Ca^{2+} concentrations. (B) Dmc1-E157D is heat unstable. D-loop reactions were staged as described in the Materials and Methods, including with or without pre-incubation of Dmc1-WT/E157D for 30 minutes at 37°C in reaction buffer without Ca^{2+} and ssDNA before proceeding to D-loop assay containing 0.5 mM Ca^{2+}. Protein acronyms and concentrations are as follows: A = RPA (0.2 μM), D = Dmc1-WT (3 μM), E = Dmc1-E157D (3 μM), T = Rdh54 (0.1 μM). Rdh54 was used in reactions to enhance D-loop yield. D-loop activity is plotted as averages ± SEM (n = 3).

Supplementary Figure S2

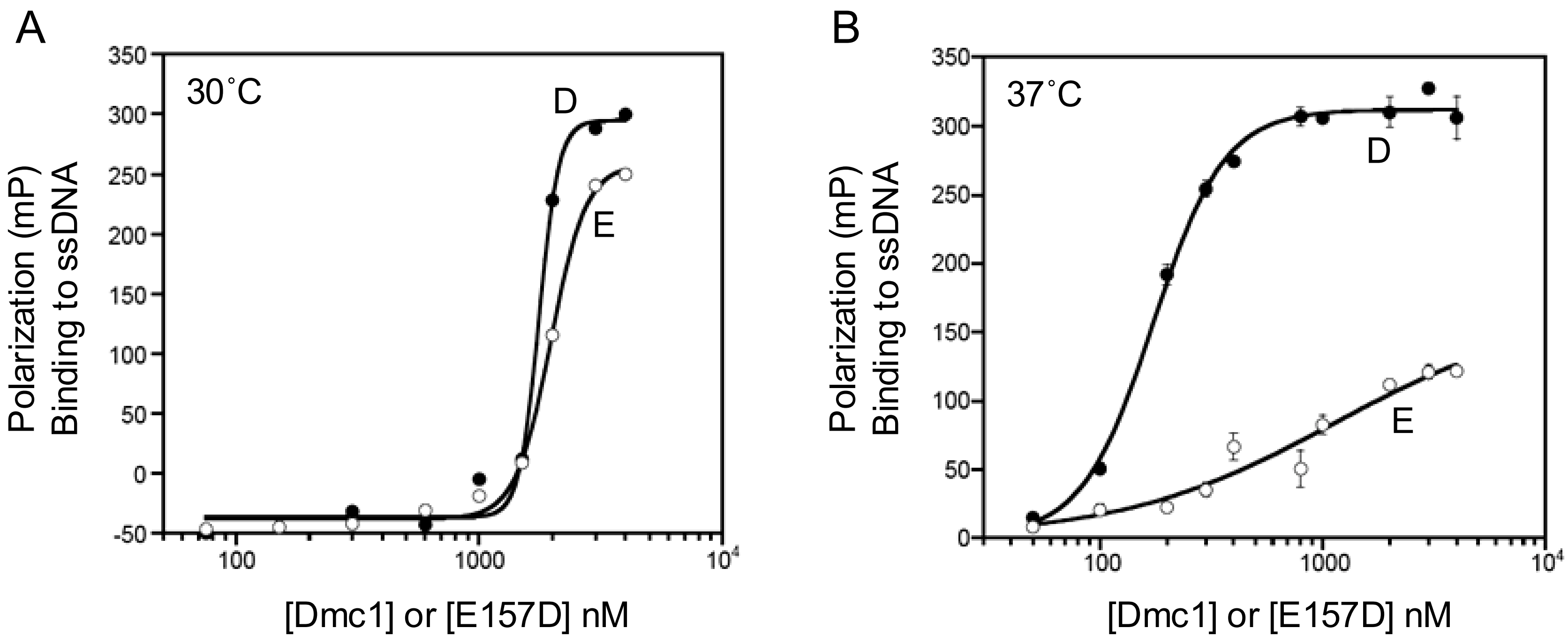

Supplementary Figure S2. Dmc1-E157D ssDNA binding is heat sensitive but Dmc1-WT ssDNA binding is not. SsDNA binding was performed with ATP and determined by fluorescence polarization (FP). (A) ssDNA binding at 30˚C. (B) ssDNA binding at 37˚C. Reaction buffer contains 70 μM Ca^{2+}. The error bars are plotted as averages from three reactions ± SEM (n ≥ 3).

Supplementary Figure S3

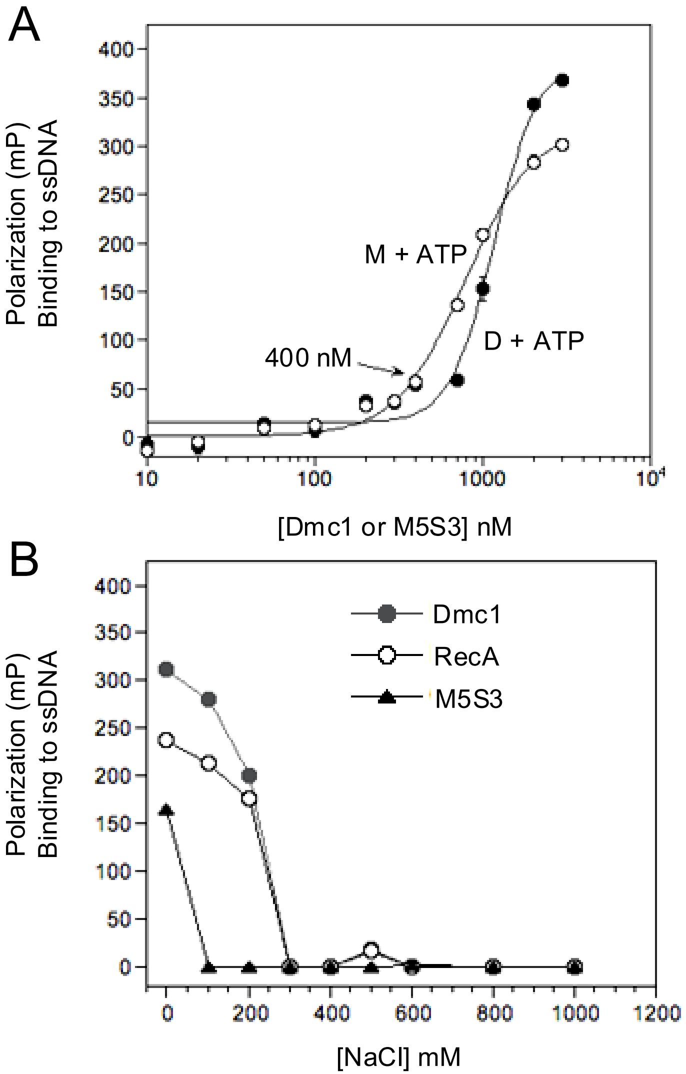

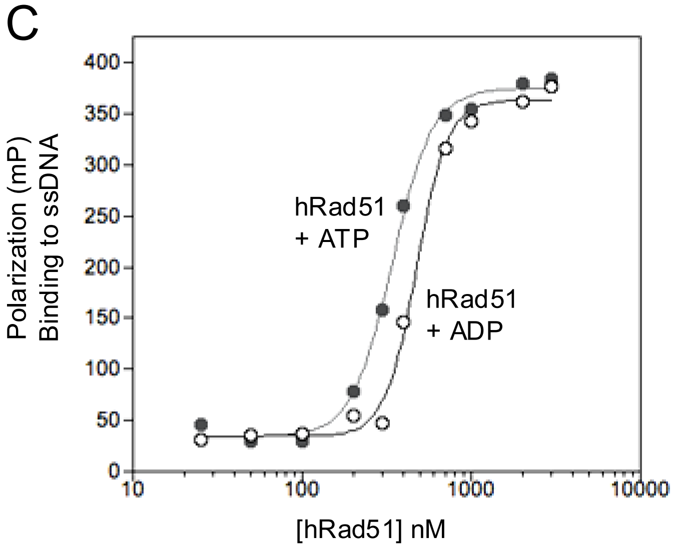

Protein	K_D (nM)
hRad51 + ATP	342 ± 10
hRad51 + ADP	476 ± 20

Supplementary Figure S3. Florescence polarization on protein binding to ssDNA. (A) Both Dmc1 (abbreviated D) and Mei5-Sae3 (abbreviated M) bind ssDNA. At 400 nM Mei5-Sae3, a very low FP signal is observed, thus this concentration was chosen to be used in subsequent ssDNA binding reactions with Dmc1. (B) Salt concentration affects protein binding to ssDNA. Increasing concentration of NaCl decreases the binding of Dmc1, RecA, and Mei5-Sae3 to ssDNA (0.25 µM-nt). However, significant binding remains for Dmc1 (90%) and RecA (90%) when NaCl is 100 mM, but no apparent binding remains for Mei5-Sae3 (at 400 nM) when NaCl exceeds 100 mM. Therefore, 100 mM NaCl was chosen to be used in ssDNA binding reactions to determine the effect of Mei5-Sae3 on Dmc1 or RecA in Figure 3. (C) Human Rad51 binds ssDNA with higher affinity with ATP than with ADP.

Supplementary Figure S4

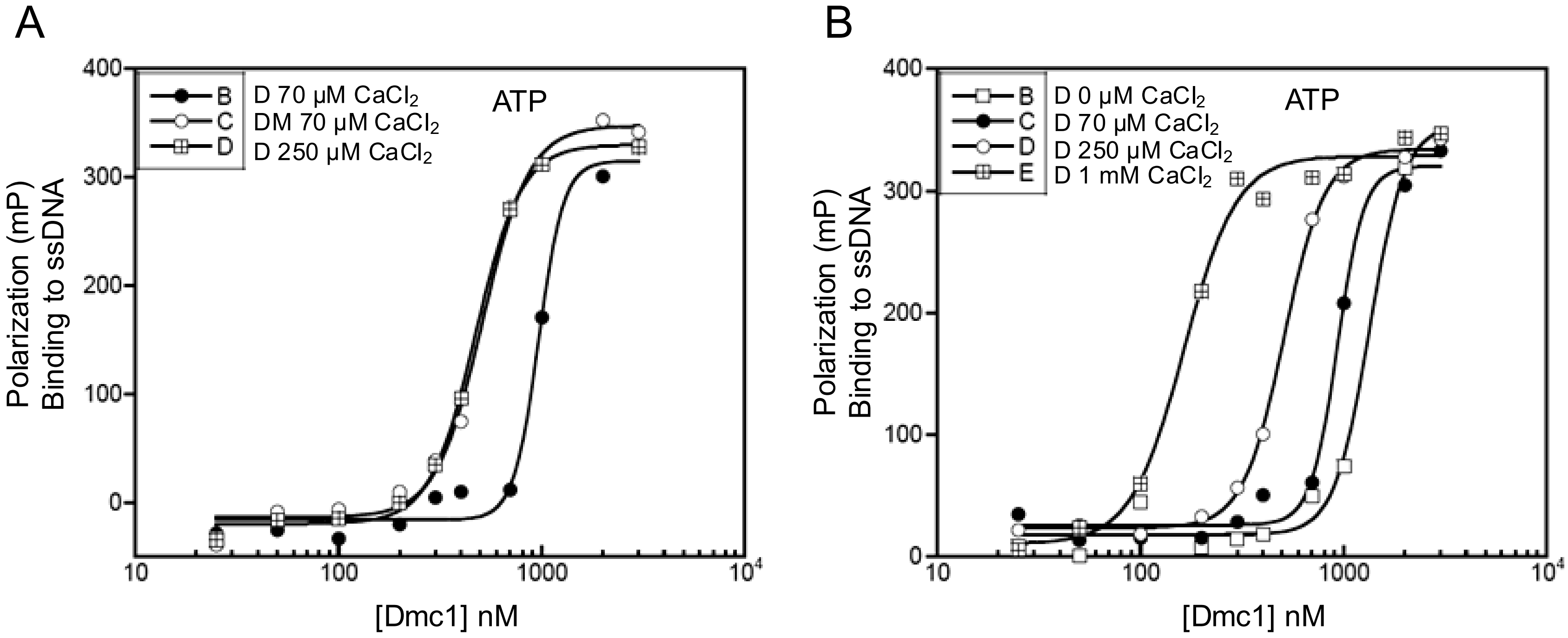

Protein	$[CaCl_2]$ µM	K_D (nM)
D	70	966 ± 32
D + M	70	512 ± 25
D	250	472 ± 14

$[CaCl_2]$ µM	K_D (nM)
0	1,341 ± 113
70	933 ± 31
250	508 ± 12
1,000	164 ± 13

Supplementary Figure S4. Ca^{2+} enhances Dmc1-WT ssDNA binding. SsDNA binding was determined by fluorescence polarization (FP). The apparent dissociation constant (K_D) of Dmc1 (abbreviated D) binding to ssDNA was determined from the plots. (A) 250 µM Ca^{2+} has the same effect on Dmc1 ssDNA binding as with Mei5-Sae3 (abbreviated M) and 70 µM Ca^{2+}. Note: The Mei5-Sae3 curve shown here is duplicated from Figure 3B. (B) Increasing Ca^{2+} concentration increases the binding affinity of Dmc1 to ssDNA as reflected by decreasing apparent K_D.

Supplementary Figure S5

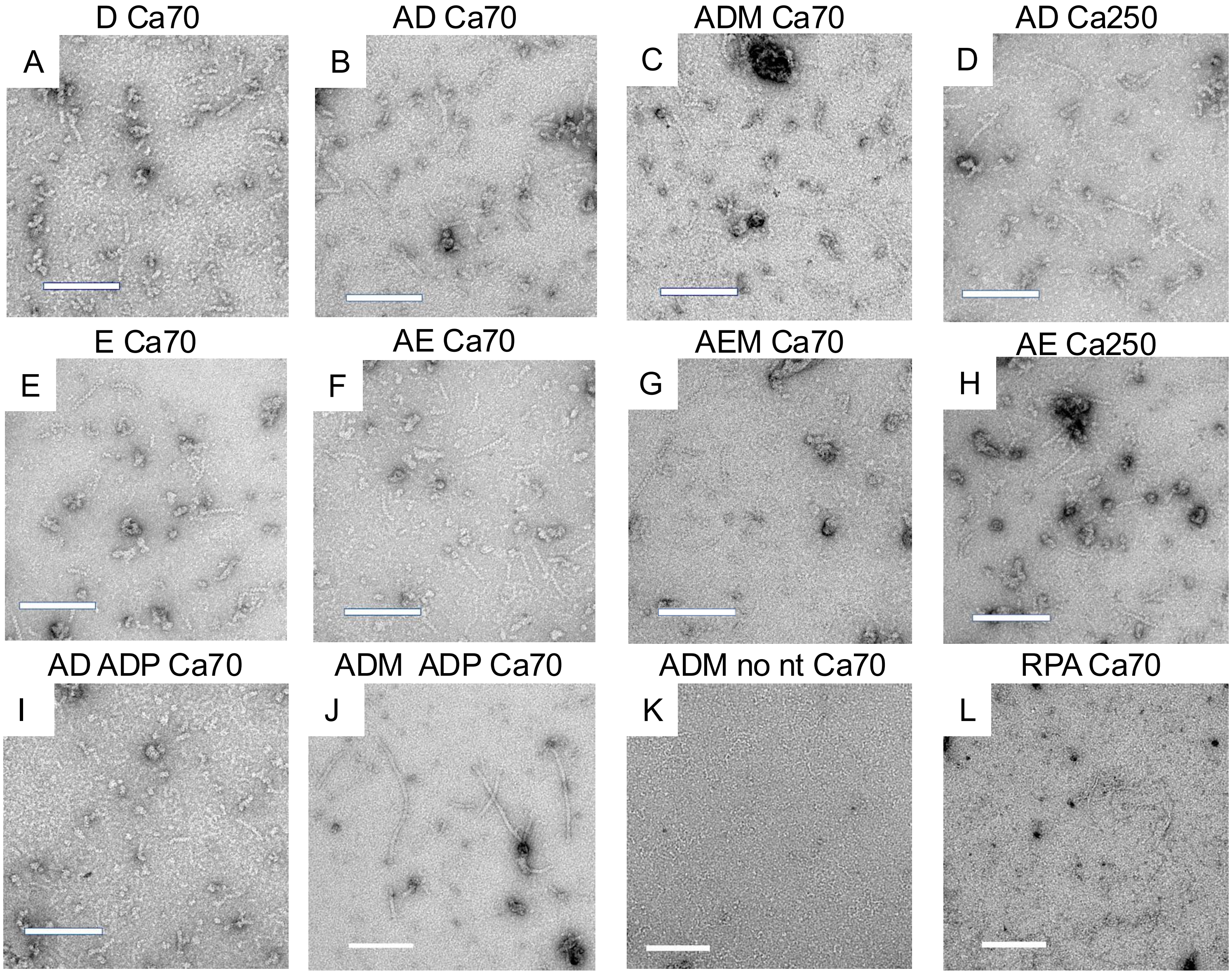

Supplementary Figure S5. Representative electron microscopy (EM) images. EM images were taken at the magnification of 25,000. White scale bar represents 200 nm. The reaction buffer for all reactions contained ATP unless otherwise specified. The conditions and proteins used in each panel are shown above the panel. Protein acronyms are: A = RPA, D = Dmc1-WT, E = Dmc1-E157D, M = Mei5-Sae3, no nt = no nucleotide cofactor added, Ca70 = 70 μM Ca^{2+}, Ca250 = 250 μM Ca^{2+}.

Supplementary Figure S6

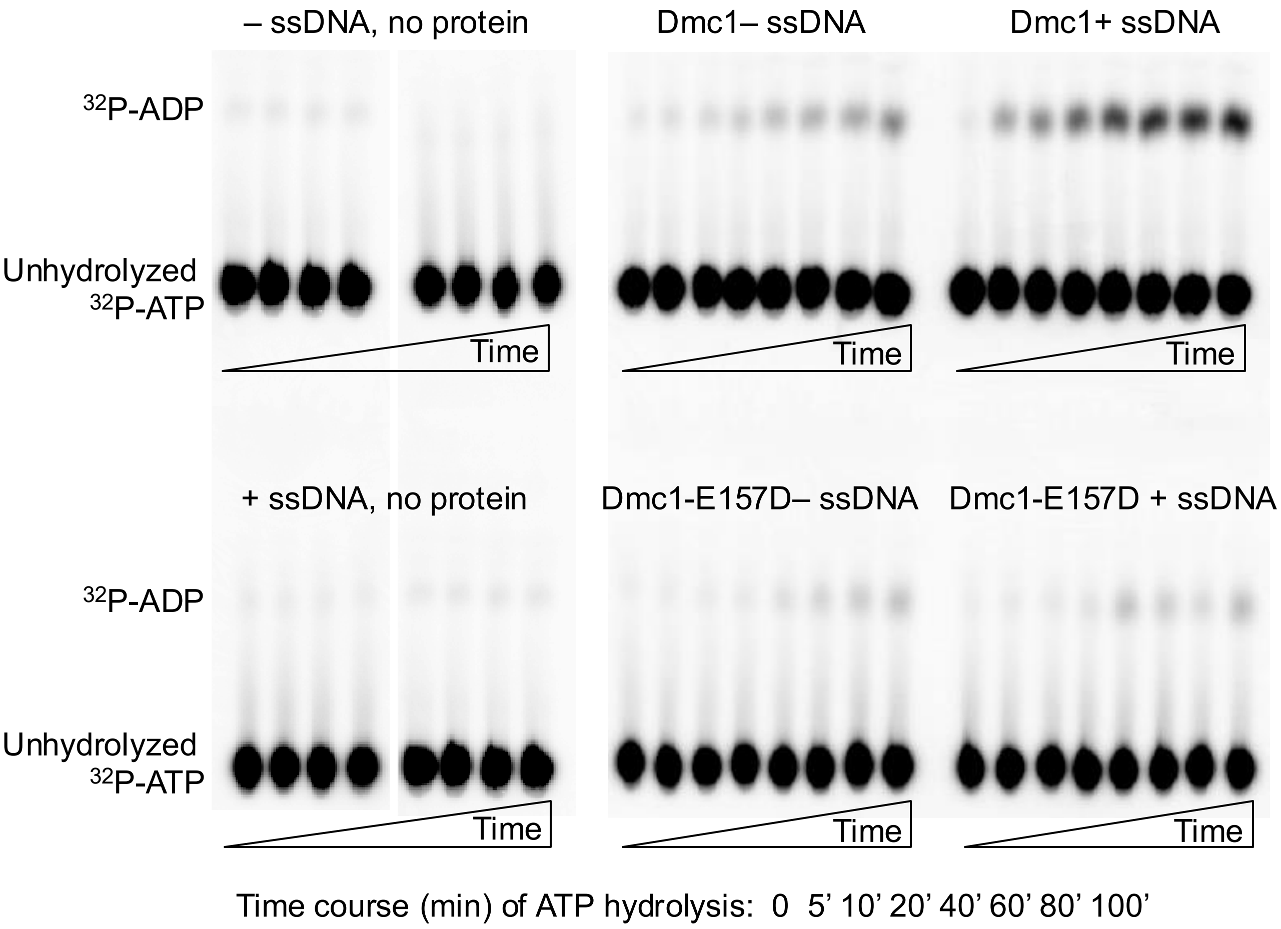

Supplementary Figure S6. The time course of ATP hydrolysis was monitored by thin layer chromatography (TLC). The original TLC chromatogram images for Figure 5C are presented here.

Supplementary Figure S7

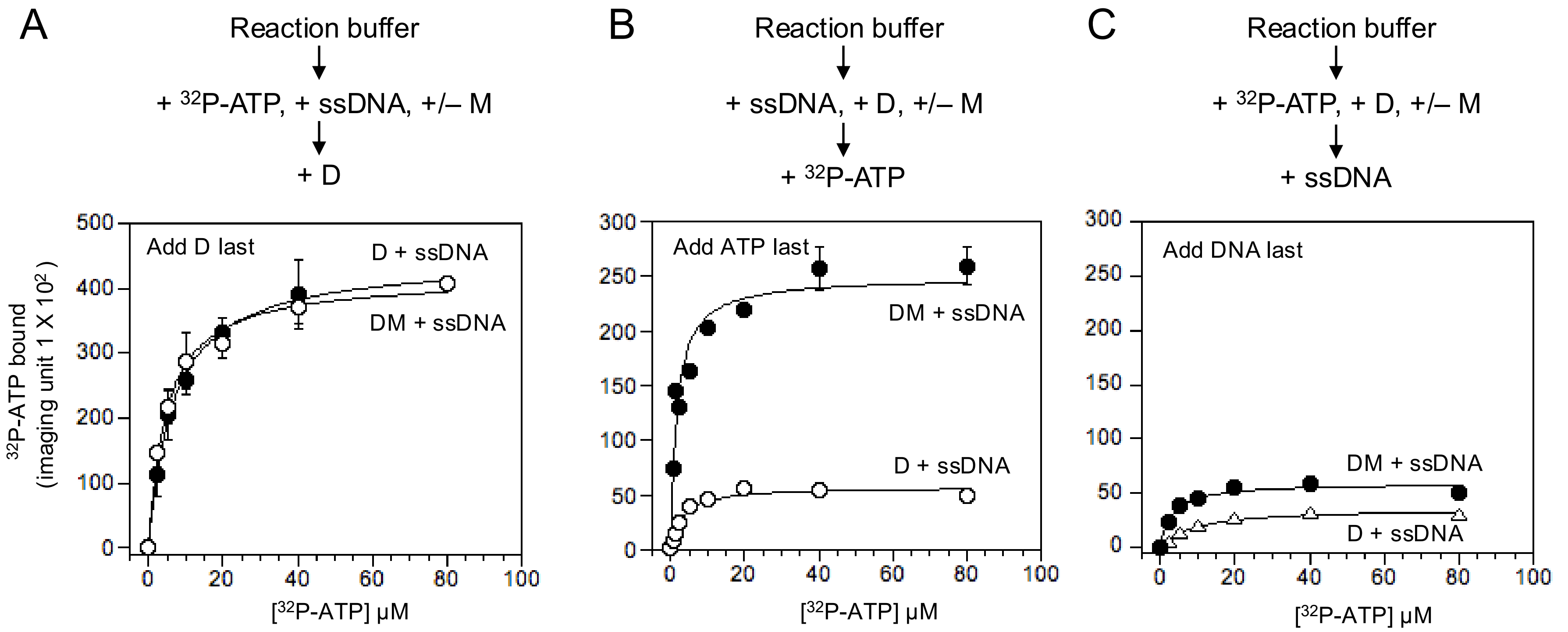

Supplementary Figure S7. The order of addition of Dmc1, ATP, and DNA in the ATP binding assay affects ATP binding by Dmc1. In a filter ATP binding assay, either Dmc1, ATP, or DNA was added last to a reaction mixture that contains all other components. (A) When Dmc1 (abbreviated D) is added last, it binds ATP/DNA to the same extent as with Mei5-Sae3 (abbreviated M). (B) When ATP is added last, Mei5-Sae3 enhances Dmc1 binding to ATP/DNA by about 4-fold. (C) When DNA is added last, Dmc1 cannot bind ATP effectively and Mei5-Sae3 cannot stimulate it. The error bars are plotted as averages from three reactions ± SEM ($n \geq 3$).

Supplementary Figure S8

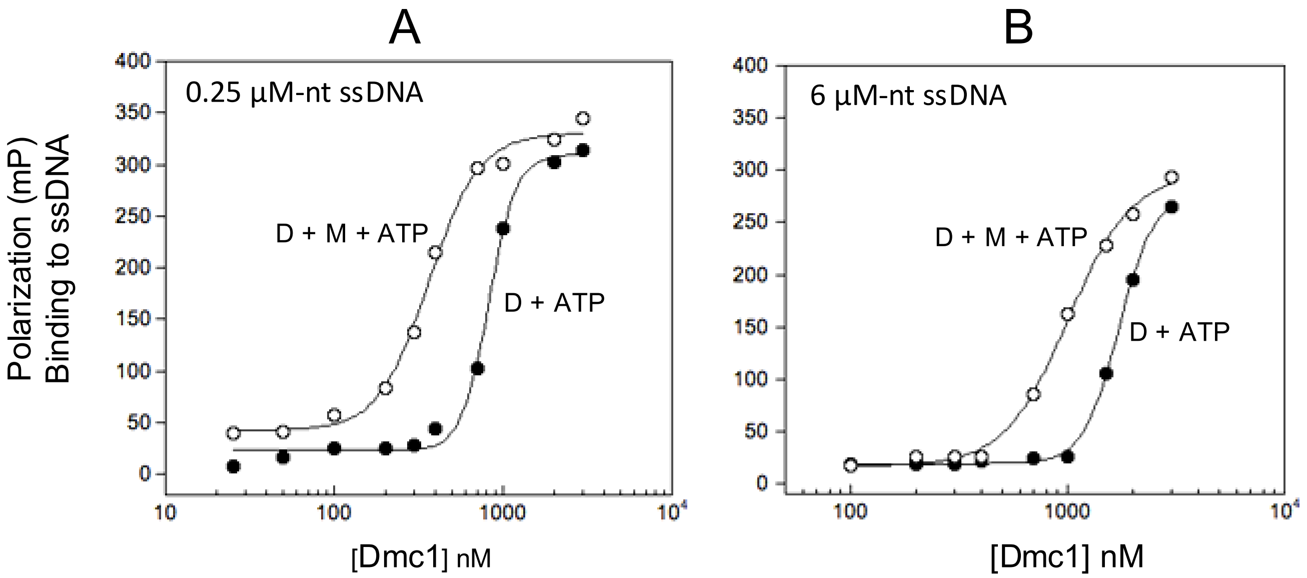

Supplementary Figure S8. Binding of Dmc1 under ATPase assay conditions shows binding is nearly saturated in both the presence and absence of Mei5-Sae3 at 3 µM Dmc1, the concentration used for the ATPase assays. Mei5-Sae3 was used at a concentration of 400 nM. (A) Data copied from Figure 3B to facilitate comparison. These results were obtained at 0.25 µM-nt ssDNA and 1 mM ATP. (B) Analysis of Dmc1 binding activity under ATPase assay conditions of 6 µM-nt ssDNA and 100 µM ATP.

Supplementary Figure S9

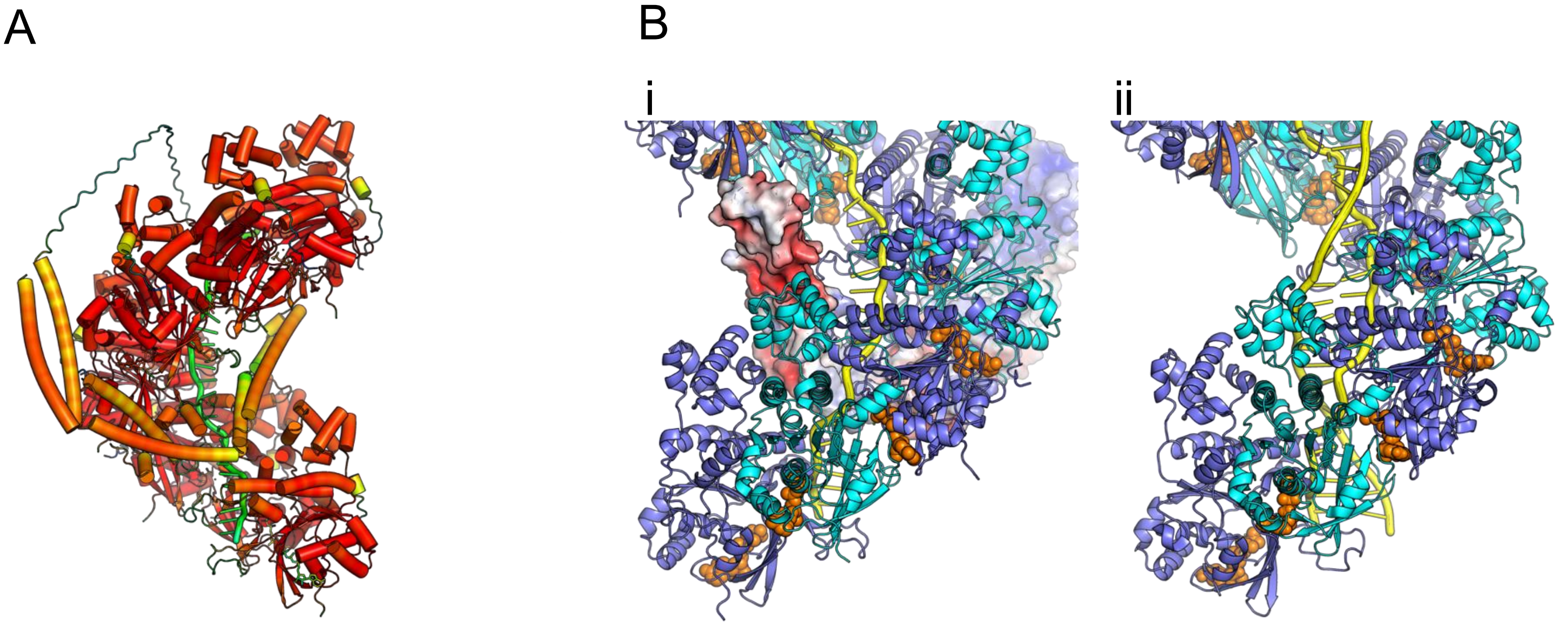

Supplementary Figure S9. Additional Model Details. (A) The same AlphaFold3 model of the Dmc1-ssDNA-ATP-Mei5-Sae3 complex as shown in Figure 8C, in the same orientation, but colored according to per-residue model confidence (red = highest; blue = lowest). The lowest confidence is for the presumably disordered N-terminal tail of Mei5, shown as an extended structure at the top left. Although confidence in the DNA modeling was surprisingly low (green) its placement closely matches that seen in experimental structures. (B) Closeup views of the same model with Mei5-Sae3 (i) and experimental structure (ii) as shown in Figure 8C, but rotated ~180 around the vertical axis. Mei5-Sae3 is shown in surface the Mei5-Sae3 surface is shown colored according to electrostatic potential (red = -5kBT, blue = +5 kBT) calculated using the ABPS plugin for PyMOL. Note the negatively charged (red) surface of Mei5-Sae3 lies where the second DNA strand lies in the duplex-bound structure (83).
